# Imaging the onset of oscillatory signaling dynamics during mouse embryo gastrulation

**DOI:** 10.1242/dev.200083

**Published:** 2022-07-11

**Authors:** Henning J. Falk, Takehito Tomita, Gregor Mönke, Katie McDole, Alexander Aulehla

**Affiliations:** 1 Developmental Biology Unit, European Molecular Biology Laboratory, 69117 Heidelberg, Germany; 2 Division of Cell Biology, MRC Laboratory of Molecular Biology, Cambridge CB2 0QH, UK

**Keywords:** Light-sheet microscopy, Notch signaling, Segmentation clock, Lunatic fringe, Period gradient, Gastrulation

## Abstract

A fundamental requirement for embryonic development is the coordination of signaling activities in space and time. A notable example in vertebrate embryos is found during somitogenesis, where gene expression oscillations linked to the segmentation clock are synchronized across cells in the presomitic mesoderm (PSM) and result in tissue-level wave patterns. To examine their onset during mouse embryo development, we studied the dynamics of the segmentation clock gene *Lfng* during gastrulation. To this end, we established an imaging setup using selective plane illumination microscopy (SPIM) that enables culture and simultaneous imaging of up to four embryos (‘SPIM- for-4’). Using SPIM-for-4, combined with genetically encoded signaling reporters, we detected the onset of Lfng oscillations within newly formed mesoderm at presomite stages. Functionally, we found that initial synchrony and the first ∼6-8 oscillation cycles occurred even when Notch signaling was impaired, revealing similarities to previous findings made in zebrafish embryos. Finally, we show that a spatial period gradient is present at the onset of oscillatory activity, providing a potential mechanism accounting for our observation that wave patterns build up gradually over the first oscillation cycles.

## Abstract (precis)

**Summary:** A versatile light-sheet imaging setup enabling simultaneous live imaging of multiple mouse embryos for 48 h, an approach that offers insight into the onset of oscillatory signaling dynamics and the segmentation clock.

## INTRODUCTION

During embryonic development, dynamic signals control and coordinate tissue patterning and morphogenesis. The dynamics of cellular signaling provide versatility to encode information, employing not only the intensity of a stimulus, but also its duration, timing and relative change (Levine et al., 2013; Purvis and Lahav, 2013; Sonnen and Aulehla, 2014).

In the context of vertebrate segmentation, dynamic Notch signaling results in rhythmic oscillatory gene expression in the unsegmented presomitic mesoderm (PSM). This molecular clock has been associated with the sequential formation of somites (Palmeirim et al., 1997). One of the most striking features of the segmentation clock relates to its spatiotemporal coordination among PSM cells. In all species studied, coherent waves of signaling activity sweep across the PSM, from the posterior end of the embryo towards the anterior PSM, where the next somite boundary forms. (Aulehla et al., 2008; Delaune et al., 2012; Masamizu et al., 2006; Soroldoni et al., 2014; Takashima et al., 2011). The appearance of these periodic wave patterns is thought to be linked to an underlying period gradient, i.e. oscillations are fastest at the posterior end and gradually slow down as cells reach the anterior PSM, before oscillations halt and segment formation ensues (Giudicelli et al., 2007; Gomez et al., 2008; Palmeirim et al., 1997; Shih et al., 2015; Tsiairis and Aulehla, 2017). These period differences cause cells to gradually shift their oscillation rhythm (i.e. their oscillation phase) relative to each other and can hereby lead to the appearance of ‘phase waves’ that sweep across the PSM.

It has previously been shown that the maintenance of synchronized wave patterns requires Notch signaling-dependent intercellular communication (Delaune et al., 2012; Horikawa et al., 2006; Jiang et al., 2000; Okubo et al., 2012; Ozbudak and Lewis, 2008; Riedel-Kruse et al., 2007; Tsiairis and Aulehla, 2017). However, far less is known about how synchrony and phase waves are established in the first place. Studies in chick and zebrafish embryos showed that the onset of synchronous segmentation clock oscillations occurs early during gastrulation, preceding segment formation (Jouve et al., 2002; Riedel-Kruse et al., 2007). Interestingly, studies in zebrafish indicated that the onset of synchronous signaling occurs even when Notch signaling mediated cell-to-cell coupling was experimentally inhibited (Jiang et al., 2000; Riedel-Kruse et al., 2007). Along similar lines, in chick embryos, the earliest signaling waves have been suggested to occur in the absence of a propagating intercellular signal (Jouve et al., 2002).

However, the dynamics and mechanism underlying the origin of these earliest spatiotemporal signaling waves during gastrulation and whether these are preceded, and hence possibly caused, by an underlying period gradient, remain unknown. This is also due to the technical challenges associated with obtaining real-time imaging quantifications of the onset of segmentation clock oscillations during gastrulation, which so far have not been obtained in any species.

Here, we tackled these challenges in the mouse model. Mouse gastrulation stages are characterized by a complex three-dimensional egg-cylinder shape, extensive growth and high photosensitivity (Garcia et al., 2011; Ichikawa et al., 2013; Nowotschin et al., 2010). Live imaging of mouse gastrulation was previously achieved using selective plane illumination microscopy (SPIM), which is especially suitable for large, photosensitive specimen because of its good optical sectioning capabilities at low light doses (Ichikawa et al., 2013; Udan et al., 2014; McDole et al., 2018).

Our goal was to combine SPIM-imaging with a perfusion-culture system that enables tunable control over environmental conditions, in order to match these to the requirements of mouse embryos from gastrulation to organogenesis stages. In addition, we established a setup that offers the possibility to perform SPIM imaging of up to four samples simultaneously, in order to facilitate quantitative and mechanistic studies. We used this setup, combined with genetic and pharmacological functional perturbations, to investigate the role of Notch signaling at the onset of signaling oscillations and in spatiotemporal signaling wave patterns during mouse gastrulation.

## RESULTS

### Customized setup enables live light-sheet imaging of mouse gastrula for >40 h

**Fig. 1.**
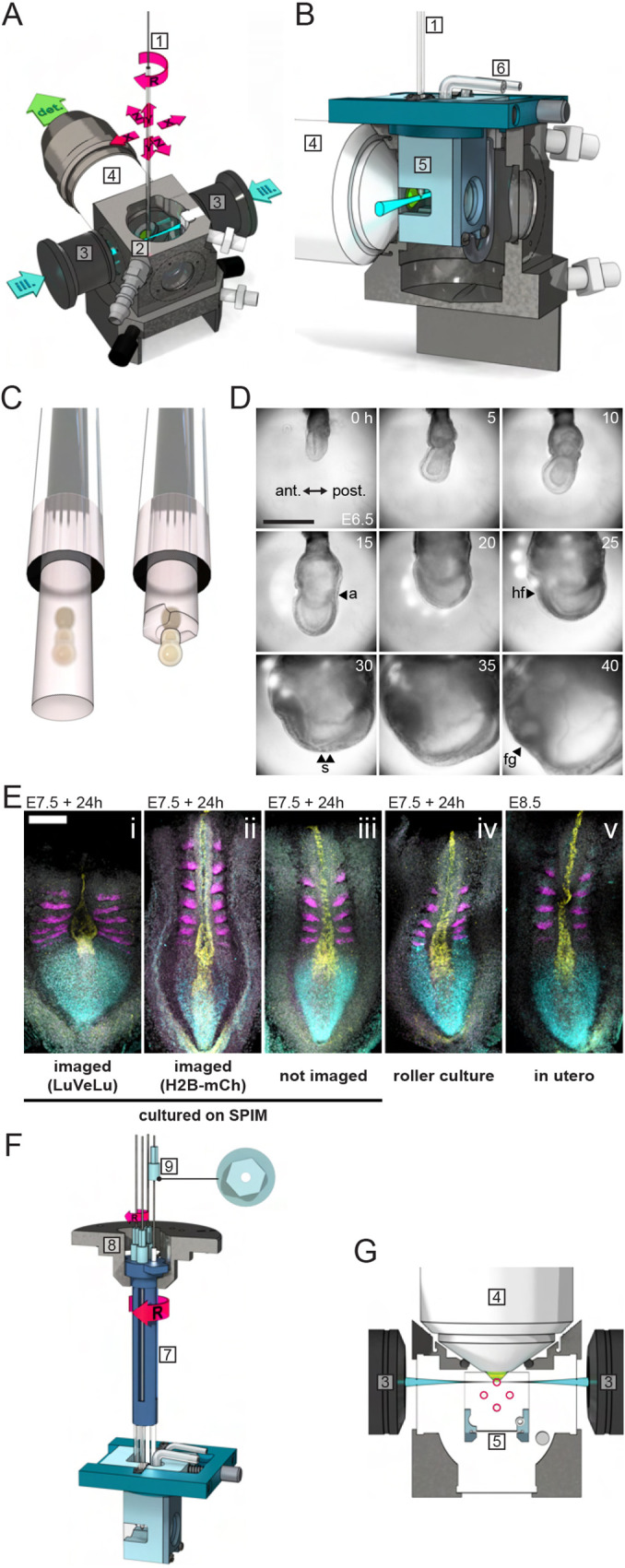
**Light-sheet microscopy for multi-sample live imaging of mouse embryos from gastrulation to organogenesis.** (A) Imaging chamber and objective configuration of the Lightsheet Z.1 (Zeiss) with two illumination light-sheets (ill.) and one detection light path (det.). The sample-containing capillary (1) is suspended from the top into the imaging chamber (2) and orientated with respect to the illumination (3) and detection (4) objectives (degrees of freedom indicated by red arrows; XYZ, translation; R, rotation). (B) Vertical section view through the sample chamber with inserted embryo culture chamber (5). The embryo culture chamber consists of a chamber lid (dark blue) and the chamber body (light blue) with membrane-covered windows for the light paths. It is connected via tubes (6) to the closed-cycle perfusion system of the microscope (Fig. S1). (C) For mounting, embryos were first agarose embedded in a capillary (left), then the agarose was peeled off around the embryo, leaving only the ectoplacental cone embedded to allow growth during culture (right). (D) Bright-field images of an embryo developing on the customized light-sheet microscope (anterior towards the left). The experiment starts at mid-streak stage at E6.5. Morphological features are the allantois (a), the head fold (hf), somites (s) and foregut pocket (fg). Scale bar: 500 μm. (E) Examples of embryos after culture on the microscope: (i) LuVeLu reporter imaging (*z*-stacks with 7.5 μm spacing, 100 slices per sample, 300 ms exposure time with 2.0% 50 mW 514 nm laser and 10 min imaging interval. *n*=6); (ii) R26-H2BmCherry reporter imaging (*z*-stacks with 7.5 μm spacing, 120 slices per sample, 200 ms exposure time with 1.2% 20 mW 561 nm laser and 10 min imaging interval. *n*=4); (iii) cultured on microscope without imaging (*n*=9); (iv) roller culture (*n*=29); and (v) *in utero* developed control embryos dissected at E8.5 (*n*=12). For all embryos, a hybridization-chain reaction (HCR)-based *in situ* mRNA hybridization was performed for following genes: *Msgn1* (cyan), a marker for presomitic mesoderm; *Shh* (yellow), expressed in notochord and the floorplate of the neural tube; and *Uncx4.1* (magenta), marking the anterior-posterior subdivisions of the somites. Scale bar: 200 μm. Brightness and contrast were set identically for samples i, iii, iv and v, but adjusted separately for ii. (F) SPIM-for-4 multi-sample holder. Four sample capillaries are positioned in the sample holder (7), which is mounted on the Z.1 stage using the standard sample holder disc for syringes (8). A capillary cap is glued to the top of each capillary (9; in top view on the right). With a hex key, each capillary can be turned individually around its long axis (small R), while the rotation drive of the microscope stage allows for switching between samples (large R). (G) Horizontal section view through the imaging chamber in the plane of the light path. Sample capillaries (red circles) in the SPIM-for-4 multi-sample holder do not interfere with the light paths during imaging and the holder is compatible with the embryo culture chamber. CAD drawings of Z.1 microscope parts are courtesy of Carl Zeiss Microscopy.

With the goal of performing long-term mouse embryo imaging, covering the onset of gastrulation at embryonic day (E) 6 to organ and somite formation on E8, we customized a culture system and mounting method for early post-implantation embryos on a commercially available light-sheet microscope (Lightsheet Z.1, Zeiss; Fig. 1A). First, we introduced an additional, smaller culture chamber into the standard Z.1 imaging chamber (Fig. 1B) to reduce volume and surface of the medium. Second, in order to generate a controlled, gradient-free gas distribution within the medium column, we implemented a closed-cycle perfusion system connected to the embryo culture chamber. The medium was constantly pumped through a gas equilibration chamber before entering the small embryo culture chamber (Fig. S1). Finally, we adjusted the mounting method in order to accommodate for the extensive growth of the embryo from about 300 μm to >2 mm in diameter, which occurs during 2 days in gastrulation stages. We embedded only the ectoplacental cone at the proximal tip of the embryo in agarose gel, while the embryo itself was vertically immersed in an unconstrained manner in culture medium (Fig. 1C). This enabled unrestricted growth and expansion of the embryo and, as an additional benefit, the light path was not further impeded by a gel or tube.

Using this modified mounting and culture setup, we successfully cultured and imaged embryos for 40 h, from early gastrulation at E6.5 up to the 10-somite stage at E8.5 (Fig. 1D, Movie 1). The quality of the culture outcome on SPIM, in terms of developmental progression, morphology and patterning, was assessed by comparing SPIM-imaging of LuVeLu reporter embryos (Fig. 1Ei), SPIM-imaged embryos expressing the bright nuclear marker H2BmCherry (Fig. 1Eii, Movie 2), embryos cultured on the SPIM setup that were not imaged (Fig. 1Eiii), embryos cultured in roller culture (Fig. 1Eiv) and freshly dissected E8.5 embryos (Fig. 1Ev). Cultured embryos from all four experimental groups progressed to early somite stages (i.e. 2-11 somites, Fig. S2) within 24 h of culture, indicating no major impact on developmental timing. At the morphological level, we found that embryos cultured on the SPIM setup for ∼24 h, which were not imaged, showed high similarity to those cultured in a roller culture system, as well as to embryos developed to E8.5 *in utero*. In contrast, embryos imaged for the segmentation clock reporter LuVeLu (Aulehla et al., 2008) from E7.5 to E8.5 with a time resolution of 1 frame/10 min (*z*-stacks with 7.5 μm spacing, 100 slices per sample) showed a visibly shorter, but wider, posterior body axis (*n*=6/6) compared with non-imaged or freshly dissected control embryos. These results indicate an impact of imaging-related phototoxicity that is likely caused by the high laser power needed to quantify the dynamic, yet dim, segmentation clock reporter LuVeLu. In agreement with this conclusion, we found that imaging of a bright nuclear reporter line (H2BmCherry) yielded developmental outcome similar to control non-imaged but cultured embryos (Fig. 1Eii,iii). Combined, our results show that the SPIM-imaging setup with perfusion culture enables a successful embryo culture from gastrulation to organogenesis stages, although phototoxicity effects must always be taken into account, especially when dynamic dim reporters are employed.

### SPIM-for-4: a simple device for multi-sample imaging

To increase throughput and enable comparative, quantitative studies between different samples analyzed simultaneously in the same experiment, we developed SPIM-for-4. SPIM-for-4 is a multi-sample imaging device that holds up to four samples in separate capillaries and is compatible with the Z.1 imaging modalities (Fig. 1F, Movie 3). Importantly, in contrast to other multi-sample approaches that were previously developed (de Luis Balaguer et al., 2016; Ichikawa et al., 2013), this strategy enables the adjustment of the orientation of samples after mounting on the microscope. This is an essential feature, as sample orientation has to be optimized in order to be able to take light-scattering of mouse embryos after E6.5 into account. For example, for successful imaging of dynamic reporter expression at the posterior side of an E6.5 embryo, this side has to be oriented towards the detection objective.

SPIM-for-4 is an optional add-on compatible with the embryo perfusion-culture setup described above. The configuration of sample capillaries ensures that while one sample is imaged, the other samples do not interfere with the light-sheet or the detection path (Fig. 1G). Switching between samples is accomplished by the rotation drive of the microscope stage using the standard Z.1 stage control; the switching time is ∼2 s. This multi-position imaging had no discernible effect on the positional stability of each sample (Fig. S3). Thus, the SPIM-for-4 setup allows simultaneous embryo time-lapse imaging of multiple samples without compromising data acquisition quality.

### Spatiotemporal expression dynamics of Lfng from gastrulation to onset of somitogenesis

**Fig. 2.**
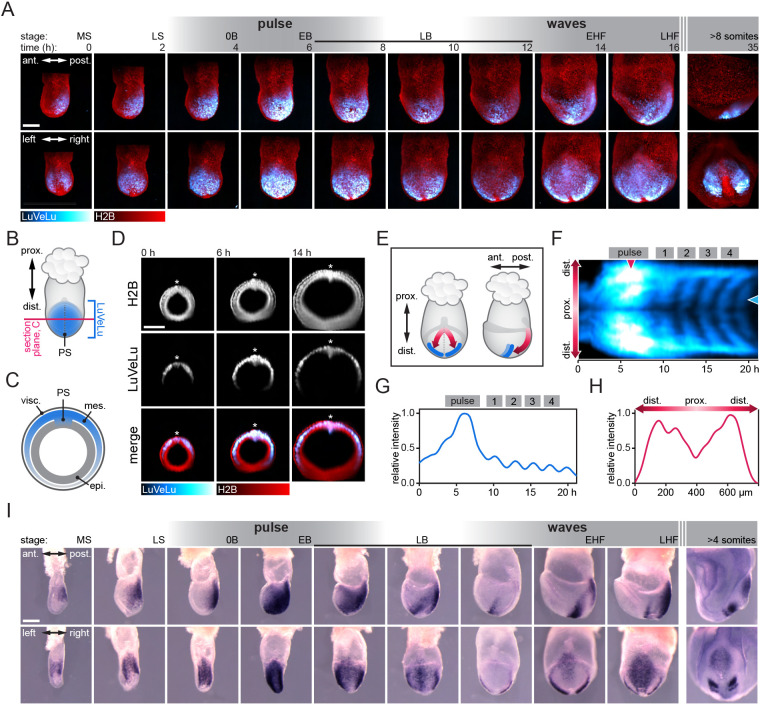
**Spatiotemporal expression dynamics of *Lfng* from gastrulation to onset of somitogenesis.** (A) Still frames of a representative LuVeLu;R26-H2BmCherry embryo imaged for 35 h starting at early gastrulation (*n*=12 similarly imaged embryos). Image stacks are shown as maximum intensity projection (MIP) from the side (top) and from posterior (bottom). LuVeLu shows an intensity pulse after 6 h and traveling waves become visible after 14 h. Embryo stages according to Downs and Davies (1993) are listed above (mid streak, MS; late streak, LS; no allantois bud, 0B; early allantois bud, EB; late allantois bud, LB; early head fold, EHF; late head fold, LHF). (B-D) Optical transverse sections through the embryo shown in A at 50% of the proximodistal extent of the LuVeLu domain (prox., proximal; dist., distal), as illustrated in B and C, are shown in D. Visceral endoderm (visc.), mesoderm (mes.) and epiblast (epi.). LuVeLu expression is localized to the PS and mesoderm. (E) Cartoon of E7.5 embryo in posterior view (left) and side view (right) to illustrate domain and directionality of early waves (red arrows) in paraxial mesoderm (PS, dotted line). Early somite boundaries are depicted in blue. (F) Intensity kymograph of LuVeLu expression of another representative embryo along the direction of waves [red double-headed arrow in E (left)] showing the pulse and the first waves. The proximal is in the center, the distal is above (left side) and below (right side). (G) Intensity time series at the proximal position indicated with the blue arrowhead in F showing pulse and waves 1-4, normalized to the maximum value of the profile. (H) Intensity profile along proximo-distal direction at time of pulse peak as indicated by the red arrowhead in F, normalized to the maximum value of the profile. (I) Reconstructed time series of *Lfng* mRNA expression between E6.5 and E8.5. The same embryos are shown in a side view (top) and from posterior (bottom). Number of samples collected for the different stages: MS, 9; LS, 6; 0B, 8; EB, 10; LB(1), 16; LB(2), 6; LB(3), 28; EHF, 2; LHF, 2; somites, 8. Scale bars: 200 μm.

We employed this customized light-sheet microscope setup to address how synchronized segmentation clock oscillations originate during gastrulation. To this end, we performed SPIM-for-4 imaging using the segmentation clock reporter LuVeLu and quantified reporter dynamics starting at E6.5, for a period of more than 35 h (Fig. 2A; Movie 4).

LuVeLu expression was first visible at E6.5 at the posterior side of the embryo. Optical transverse sections through the embryo proper revealed LuVeLu-positive cells in the primitive streak (PS) and nascent mesoderm (Fig. 2B-D). The LuVeLu expression domain progressively expanded as the mesoderm layer formed its characteristic ‘wings’ (Downs and Davies, 1993) that cover most of the egg cylinder at early (allantois) bud (EB) stage.

The first dynamic change in LuVeLu activity was detected at EB stage, when reporter activity showed a marked (i.e. twofold) increase within a ∼2.5 h time-window within the entire LuVeLu-positive domain, including PS and mesodermal wings (Fig. 2A,H). In this work, we refer to this change in expression as ‘pulse’ to distinguish its dynamics from subsequent wave patterns (see below) and to indicate that, at least with a time-resolution of 10 min between imaging frames, the rise in reporter activity occurred in a (quasi)-synchronous manner. Following this pulse, we detected the first observable LuVeLu oscillations and spatiotemporal wave patterns (Fig. 2E-G; Movie 4). We detected the first LuVeLu oscillations and waves at the late (allantois) bud (LB) stage, ∼4.5 h (267 min median; 47 min interquartile range; IQR) after the peak of the pulse. The wave patterns then repeated periodically at intervals of ∼145 min (Fig. S4, Movie 5).

To validate the LuVeLu reporter activity patterns that we detected during live imaging, we followed two complementary strategies. First, we analyzed endogenous *Lfng* mRNA expression in stage-matched mouse embryos collected between E6.5 and E8.5 (Fig. 2I). We found *Lfng* mRNA patterns reminiscent of LuVeLu patterns described above, with evidence for a widespread marked increase of *Lfng* mRNA expression within PS and mesoderm wings at EB stage. In addition, comparison of mRNA patterns across littermates showed variable expression patterns, indicative of traveling waves in late allantois bud and head-fold stages (Fig. 2I). Second, to further validate the transgenic LuVeLu reporter, which contains a PSM-specific cis-regulatory sequence upstream of Lfng (Morales et al., 2002; Cole et al., 2002), we generated a knock-in Lfng reporter expressed from the endogenous *Lfng* locus (LfngT2A3xVenus, Fig. S5). We found that, although overall reporter intensity was lower, the LfngT2A3xVenus reporter recapitulated LuVeLu expression dynamics, with clear evidence for pulse and wave-patterns in nascent mesoderm cells (Fig. S5, Movie 6).

### Disruption of Notch signaling does not prevent initial synchronous signaling

**Fig. 3.**
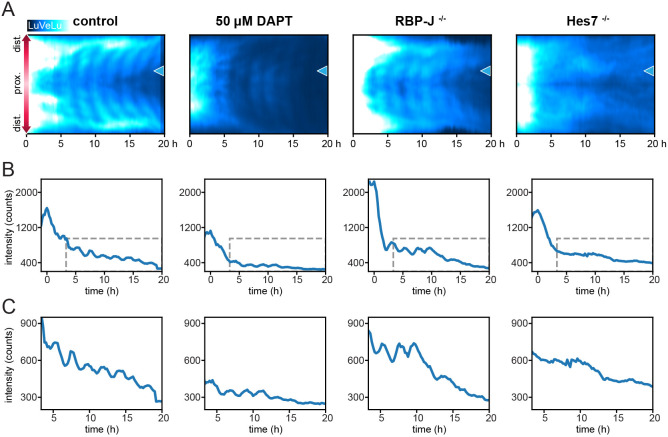
**Genetic and pharmacological disruption of Notch signaling during establishment of the segmentation clock.** (A) Representative LuVeLu fluorescence intensity kymographs of control (*n*=11), embryos treated with 50 μM DAPT from MS-LS stage onwards (*n*=7), and *Rbpj*^−/−^ (*n*=5) and *Hes7* ^−/−^ (*n*=6) mutant embryos are shown. Kymographs were drawn along the direction of wave propagation as depicted in Fig. 2E. Time point zero is set to the peak of the pulse for all panels in this figure. Brightness and contrast were set identically for all samples. (B) Intensity profiles at the proximal position indicated by a blue arrowhead in A. (C) Expanded plots of intensity profiles inside the gray dashed boxes indicated in B, depicting the oscillations that occur after the pulse.

We next performed pharmacological and genetic functional experiments to test the role of Notch signaling during the onset of the pulse and spatiotemporal wave patterns. To this end, we first treated embryos with the gamma-secretase inhibitor DAPT, which prevents ligand-activated Notch signaling (Dovey et al., 2001), and quantified the effect on LuVeLu reporter dynamics. Embryos were cultured in 50 μM DAPT from 6.5 dpc [mid-streak (MS)/late streak (LS) stages] onwards and imaged starting at E7.5 [early bud (EB) stage] until E8.5. We found that upon DAPT treatment, the LuVeLu reporter showed a widespread synchronized pulse of activity in mesoderm and PS cells at the EB stage, comparable with control embryos (Fig. 3A, Fig. S6). In embryos treated with DAPT, we detected three to seven spatiotemporal wave patterns, while subsequent oscillations were not observed and overall reporter intensity dropped to background levels (*n*=7/7; Fig. 3B,C, Movies 7, 8).

As an orthogonal, genetic approach to disrupt Notch signaling, we analyzed mutants of the core Notch-signaling transcriptional activator *Rbpj*. Genetic deletion of *Rbpj* has been previously shown to result in a fully penetrant embryonic segmentation phenotype that becomes apparent at 9.5 dpc (Oka et al., 1995; Han et al., 2002). Here, we specifically addressed the impact of *Rbpj* deletion on the pulse and the onset of segmentation clock oscillations. Unexpectedly, we found that in a large proportion of mutant *Rbpj* embryos that we collected (19/30), morphological malformations and signs of development arrest were visible already at day 6.5 dpc, which to our knowledge was not reported previously (see Materials and Methods for more details). The remaining mutant embryos (11/30) showed the expected phenotype matching previous reports, and these were used for further analysis. We performed LuVeLu SPIM-for-4 imaging in *Rbpj*^−/−^ mutant embryos and found that the synchronous pulse at the EB stage was detectable in *Rbpj*^−/−^ mutants (Fig. 3A, Fig. S6, Movie 9, *n*=5/5). Following the pulse, oscillations were visible up to a maximum of eight cycles in most mutant embryos (*n*= 4/5). To further validate this result, based on the analysis of the LuVeLu reporter, we also analyzed endogenous Lfng mRNA expression in *Rbpj*^−/−^ embryos. We found that expression levels were comparable between *Rbpj*^−/−^ and control embryos, with a clear indication of a marked increase in mRNA expression levels at the EB stage (Fig. S7), corresponding to the ‘pulse’ seen during LuVeLu imaging at these stages. Combined, our results indicate that the signaling pulse at the EB stage and, interestingly, also the first oscillations and spatiotemporal wave patterns, occur even when Notch-signaling is impaired in *Rbpj*^−/−^ embryos.

Additionally, we tested the functional role of Hes7 at the onset of LuVeLu oscillations. Hes7 has been identified as a central segmentation clock element forming a negative-feedback regulatory loop that underlies oscillatory Notch signaling activity (Bessho et al., 2003; Hirata et al., 2004). To test the role of Hes7 at the onset of oscillations during gastrulation, we analyzed LuVeLu dynamics in *Hes7*^−/−^ embryos. In *Hes7*^−/−^ mutant embryos, we detected the synchronous pulse at the EB stage (Fig. 3A, Fig. S6, Movie 10, *n*=6/6), while subsequent oscillations and wave patterns were not observed in these embryos (*n*=6/6).

Our results hence reveal that the first six to eight oscillations are present when Notch signaling is disrupted with DAPT or in *Rbpj*^−/−^ mutants. At the same time, we found clear evidence that also the first observed signaling oscillations require the core segmentation clock gene *Hes7*.

### Onset of signaling oscillations precedes formation of first somites

**Fig. 4.**
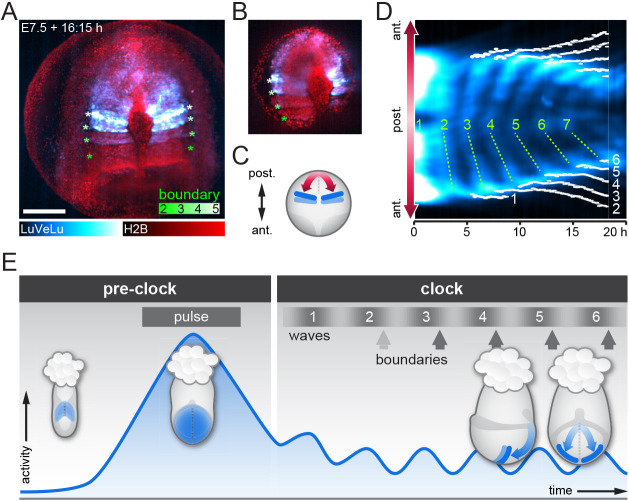
**Onset of oscillatory signaling precedes somite boundary formation.** (A) Representative LuVeLu;R26-H2BmCherry embryo imaged from distal side (posterior top) to monitor segment boundary formation; a maximum intensity projection is shown (*n*=10). Boundaries are visible as gaps between nuclei (color-coded asterisks). Scale bar: 200 μm. (B) Single *z*-plane of the dataset shown in A to better visualize the gaps between segments. (D) Kymograph of the embryo shown in A and B along the direction of wave propagation as depicted in C (ant., anterior; post., posterior). The position of each segment boundary was manually traced through the time series; traces are shown in white. Waves are numbered in green; dotted lines marking the waves were manually placed for visualization. The first segment boundary forms when the second wave is seen distally at the node region. In the embryo shown, as in the majority of analyzed samples (*n*=7/10), the first boundary disappears while all later boundaries persist. (E) Scheme to summarize the patterns of LuVeLu activity in the gastrulating embryo. During the pre-clock stage, we find a widespread signaling pulse in PS and mesoderm wings at EB stage, which is clearly distinct from later oscillatory activity by its amplitude and duration (Fig. 2G, Fig. S4). The first oscillation and wave marks the onset of the segmentation clock (‘clock stage’). Although a transient segment boundary is formed at the end of the second wave, stable segment boundaries start to form with the third wave.

To analyze the onset of signaling oscillations and wave dynamics in relation to the start of somite formation, we performed simultaneous light-sheet imaging using the LuVeLu reporter in combination with the nuclear marker H2BmCherry. Mesoderm segmentation was visualized as clefts between the otherwise evenly distributed nuclei. During the first oscillation and wave cycle, we found no evidence for segmentation using these markers. At the time the second wave pattern was detected distally at the level of the node, we found that a cleft formed at this position. Yet this initial boundary was transient and disappeared within the first five oscillation cycles in the majority of analyzed embryos (*n*=7/10; Fig. 4A-D, Movie 11). Stable and clear segment formation occurred with the third oscillation cycle onwards. Hence, several rounds (i.e. two) of signaling oscillations and wave patterns precede the formation of first somites. This finding during mouse gastrulation qualitatively resembles previous reports made in chick and zebrafish, showing that two (in chick) to five (in zebrafish) oscillations precede the first somite formation (Jouve et al., 2002; Riedel-Kruse et al., 2007).

### A period gradient accompanies the start of the segmentation clock

In order to investigate the dynamic origin of wave patterns, we asked next whether a period gradient is present at the onset of oscillations. An oscillation period gradient can directly result in the appearance of traveling wave patterns, i.e. phase waves, owing to the gradual increase in phase-shift between regions with faster versus slower oscillations.

During somitogenesis, the presence of a period gradient along the body axis has been identified as a key characteristic in several species. In mouse, zebrafish and chick embryos, it was found that segmentation clock oscillations are fastest in the posterior PSM and slower towards the anterior PSM (Giudicelli et al., 2007; Gomez et al., 2008; Palmeirim et al., 1997; Shih et al., 2015; Tsiairis and Aulehla, 2017). However, whether or not a period gradient already exists at the onset of oscillations is so far unknown.

**Fig. 5.**
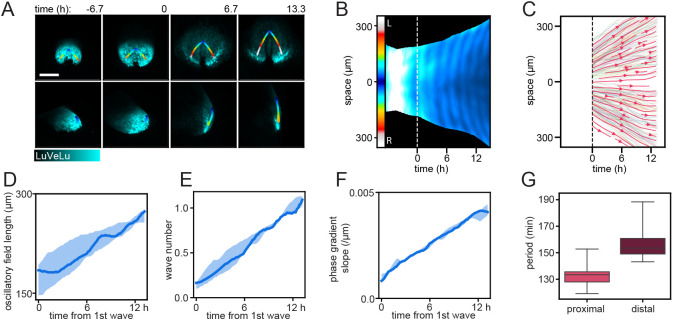
**Phase and period dynamics of *LuVeLu* oscillations during the first waves.** (A) Still frames of a representative *LuVeLu* embryo and the line of interest following the curved surface of the mesoderm. Imaged for over 20 h starting at no allantois bud stage (*n*=5 similarly imaged embryos). Image stacks are shown as maximum intensity projection (MIP) from the posterior (top) and from side (bottom). Scale bar: 200 μm. (B) Intensity kymograph along the line of interest shown in A. The proximal region is registered at the center of the kymograph, while the left and right distal ends extend above and below, respectively. Timepoint zero is set when the first wave is observed, represented by the white dashed line. (C) Streamline plot showing flow of cells, based on cell tracks obtained from four mouse embryo datasets. Cell tracks were obtained by transferring the line of interest used to generate surface kymographs. The tracks were summarized on a common coordinate system, in which the proximal region is registered at the center, while the left and right distal ends extend above and below, respectively. Each shaded line represents a single cell track (see also Fig. S9A for plots of individual samples). Cells staying within the 30 μm radius of the line of interest for more than 300 min were included for generation of streamlines. One out of eight of the entire set of cell tracks are visualized as single cell tracks. (D) Length of the LuVeLu oscillatory field measured along the line of interest following the surface of the mesoderm, from the proximal oscillation origin to the distal end. (E) Total phase shift within the mesoderm from proximal to distal, quantified as wave number q (q=1: one wave is visible within the entire mesoderm field). (F) The linear estimate of phase gradient slope, obtained by dividing the wave number by oscillatory field length at each timepoint. (G) Period measured in proximal and distal regions for ∼2-6 h after the initiation of waves, corresponding to the second and third waves. Proximal measurements were taken at the midline of the kymograph, while distal measurements were taken from the left side of the embryo at the contour paths shifted 20 pixels ≈41.8 μm toward the midline. Boxes represent the IQR, whiskers extend to minimum and maximum values. (D-F) Solid lines represent medians; shaded areas mark IQR.

To address this question, we quantified oscillation phase and period dynamics along the proximo-distal axis in gastrulating mouse embryos, taking the curved, three-dimensional geometry of the embryo into account. To this end, we first quantified LuVeLu reporter intensity using surface kymographs, which extend from the proximal wave origin to where wave propagation ends distally, following the embryo curvature (Fig. 5A,B, *n*=5, Movie 12, see Materials and Methods). In addition, to ensure that a similar set of cells is followed over time and their oscillation dynamics quantified, we also wanted to take cell movement in mesoderm tissue into account. This is especially important given the extensive growth of the mesodermal oscillating tissue during the culture (Fig. 5A,B).

For this purpose, we used the reference dataset presented by McDole et al. (2018), which provides unprecedented *in toto* single cell tracking from four gastrulation stage mouse embryos. We extracted cell movement from these reference tracking datasets, limiting the analysis to a specific area within the mesoderm that we specified based on the location of the LuVeLu surface kymographs (Fig. 5C, Fig. S9A, Movie 13). Analysis of these cell tracks showed that mesodermal cell movement at this stage can be characterized as a proportional displacement within the growing tissue, with a velocity of 0.1-0.3 μm/min (Fig. 5C).

We then defined two regions of interest (ROIs) approximating cellular trajectories, i.e. close to the wave origin and distally in the mesodermal tissue; in these ROIs, we quantified the period during the first oscillation cycles. Our results reveal that, indeed, a period gradient along the proximo-distal axis is apparent from the very first detectable oscillations, e.g. oscillations are slower by ∼20 min in the distal mesoderm compared with oscillations in cells at the wave origin (Fig. 5G, proximal: median 133 min, IQR 7.67 min, distal: median 153 min, IQR 11.7 min).

Interestingly, our quantifications also revealed a gradual build up of oscillation phase differences along the embryonic axis (quantified as wave number q; Kopell and Howard, 1973) that, over time, resulted in the appearance of traveling wave patterns, i.e. phase waves. Hence, at the onset of visible oscillations, mesoderm cells along the proximo-distal axis are essentially oscillating in-phase (q=0.16, median; 0.07, IQR) and, over time, the phase difference gradually increases so that by the 6th oscillatory LuVeLu cycle, a full wave spanning the embryonic mesoderm is detected (q=1.09, median; 0.11, IQR).

We found that, in addition to the build-up in overall phase difference within the entire tissue, the local phase difference per unit length, i.e. the phase gradient slope, also increased (Fig. 5D-F). Taking into account that cell density decreases monotonously during this timeframe (Fig. S9B), the increase in phase gradient slope provides additional independent evidence for the presence of a spatial period difference between oscillators. Combined, our results reveal that the spatial period differences along the embryonic axis are already present at the onset of detectable LuVeLu oscillatory activity and can account for the gradual build-up of spatiotemporal wave patterns during the first oscillatory cycles.

## DISCUSSION

In this study, we have investigated the onset of synchronized signaling dynamics during gastrulation in mouse embryos using SPIM imaging. To this end, we developed a perfusion-culture and multi-sample holder setup as an extension to the commercial Z.1 light-sheet microscope. This enabled us to analyze the earliest expression dynamics of the Notch target *Lfng* and to reveal the onset of synchronized signaling dynamics using real-time reporters.

### SPIM-for-4 enables multi-sample light-sheet imaging

To enable long-term live imaging of post-implantation mouse embryos, we developed a culture and mounting strategy that is readily integrated into the Z.1 framework. Unique advantages of this approach are that (1) customization is carried out without any changes to the hardware or software configuration, providing an easy option to upgrade existing commercial light-sheet microscopes; (2) the mounting strategy allows full control of embryo orientation during imaging, yet does not impede growth of the sample; and (3) the combination with the SPIM-for-4 multi-sample holder enables a substantial increase of experimental throughput of long-term imaging experiments. This modified imaging and culture setup facilitated imaging of mouse embryos for up to 2 days, starting from early gastrulation on E6.5, well into the somite formation stage (E8.5). The implementation of a perfusion culture system facilitates the adjustment, even during the imaging experiment, of the culture conditions to match stage-dependent requirements and hence could be an important additional feature compared with already established alternative methods (Ichikawa et al., 2013; Udan et al., 2014; McDole et al., 2018). Further optimization of culture conditions should aim to reduce the observed (phototoxic) imaging side effects, which we associate with the need for high laser power to visualize dim dynamic signaling reporters. The development of brighter, fast-folding reporters (Yoshioka-Kobayashi et al., 2020) will provide an additional strategy to increase the resolution, in time and space, with the goal to reveal, in future studies, single cell signaling dynamics.

Using SPIM-for-4, we were able to perform real-time quantifications of the earliest detectable segmentation clock oscillations in mouse embryos. We found the oscillation period measured in proximal paraxial mesoderm for the first waves to be ∼140 min. This is very similar to PSM oscillations measured in later (E10.5) clock stage embryos (Tsiairis and Aulehla, 2017), while earlier studies had predicted a considerable faster segmentation rate of the first anterior somites (Tam, 1981). We cannot fully resolve this apparent discrepancy but from the direct comparison of embryos from imaging experiments to control *in utero* developed embryos revealed no obvious developmental delay (Fig. 1E). We hence conclude that the signaling and patterning dynamics we quantify in culture/imaging experiments are reflecting, or at least approximating, endogenous processes occurring *in utero*.

In summary, this culture and imaging setup closely mimics the well-established embryo culture techniques, such as embryo roller culture (New and Cockroft, 1979; Tam, 1998), with the added obvious benefit to enable simultaneous real-time imaging experiments. Our embryo culture system should be readily usable to address diverse biological questions in the gastrulation-to-organogenesis stage mouse embryo, and thanks to the multi-sample holder option, even medium-scale experiments, such as mutant phenotyping, are now feasible. In addition, beyond the mouse model system, this perfusion-culture and multi-sample SPIM imaging system may also be adapted to other embryos as well as organoid studies.

### The role of Notch signaling at the onset of oscillatory signaling during gastrulation

One key finding from our study is that the first oscillations and spatiotemporal wave patterns occur both upon pharmacological (i.e. DAPT) and genetic (i.e. *Rbpj*) perturbation of canonical Notch signaling. Specifically, we found up to six oscillatory cycles and wave patterns of the LuVeLu reporter in *Rbpj*^−/−^ mutants (*Rbpj* is the key mediator of canonical Notch-signaling during gastrulation; Souilhol et al., 2015).

In addition, we found that oscillations are dependent on Hes7 at their onset and during the first cycles, and hence no spatiotemporal wave patterns are visible in *Hes7* mutant embryos. These results closely mirror previous findings made in zebrafish embryos where the first synchronous oscillations of the segmentation clock require h/E(Spl) transcriptional repressors, but do not depend on Delta-Notch signaling (Riedel-Kruse et al., 2007). At the onset of signaling oscillations, canonical Notch-signaling is dispensable in both zebrafish and mouse embryos.

At a more-detailed level, our results indicate that the LuVeLu reporter, which is driven by a 2.4 kb cis-regulatory sequence upstream of Lfng and which had been shown to be responsive to changes in Notch-signaling (Dale et al., 2006; Ferjentsik et al., 2009; Hubaud et al., 2017), receives a Notch-cleavage independent input at the onset of segmentation clock activity. In this regard, it is interesting to note that in a recent *in vitro* study using PSM cell culture methods, the YAP/TAZ pathway was linked to LuVeLu oscillatory activity (Hubaud et al., 2017). In particular, DAPT-insensitive LuVeLu oscillations were found in a condition of suppressed YAP/TAZ signaling (Hubaud et al., 2017). Currently, the *in vivo* functional role of YAP/TAZ signaling at the onset of signaling oscillations during mouse gastrulation is unknown and hence future studies are needed, for which the combination of mouse genetics with the SPIM-for-4 is a promising approach.

We found that that the earliest oscillatory LuVeLu activity is already detectable within newly formed mesoderm at the late allantoic bud stage, preceding visible segmentation of the paraxial mesoderm. The identity and fate of these earliest mesodermal cells showing oscillatory activity needs to be precisely determined in future studies and could provide important molecular insights into the long-standing questions about head mesoderm organization (Horder et al., 2010).

From a comparative perspective, our results are in qualitative agreement with previous studies made in chick and zebrafish embryos (Jouve et al., 2002; Riedel-Kruse et al., 2007). Quantitatively, the number of oscillations preceding segmentation differs between species, with five oscillations/waves observed in zebrafish, two waves in chick and the pre-clock pulse plus two waves in mouse embryos. In addition, in the above studies the oscillatory cycles were localized to presumptive mesoderm region in the epiblast, while LuVeLu reporter activity is confined to the primitive streak and formed mesoderm. So far, oscillatory activity within the epiblast region has not been reported in mouse gastrula, and future studies, including additional reporters for Wnt and Fgf signaling, will need to address whether oscillatory signaling activity in the epiblast is present in the mouse embryo.

### Onset of spatiotemporal signaling wave patterns during gastrulation

Our real-time quantifications provide new insight into the dynamics at the onset of spatiotemporal wave patterns. Wave patterns can result from numerous distinct mechanisms, i.e. waves can reflect an active transmission of a signal across space (‘trigger waves’) or can reflect localized activity that occurs time-shifted in different regions, resulting in wave-like patterns. If the underlying activity is oscillatory, these waves are termed (oscillation) ‘phase-waves’. Quantifying the dynamics of how the waves emerge can inform about which of the two fundamentally different wave mechanisms is operating. For trigger waves, a traveling front is evident from the very beginning; in phase waves, the phase shift between oscillators in different regions may build up gradually and only over time, waves become visible. Our results show a gradual build up of waves over the first oscillation cycles (Fig. 5E), suggestive of a phase wave mechanisms. The build up of oscillation phase-shift can be the result of oscillator coupling (i.e. ‘twisted states’, Wiley and Strogatz, 2006) or, more intuitively, be directly caused by spatial period differences between oscillators.

Our quantifications show that, indeed, at the onset of detectable oscillatory activity, a period gradient already exists. The fastest oscillations occur at the proximal end, where waves originate. This period gradient precedes and can hence underlie the gradual build-up of spatiotemporal wave patterns that become visible during the first oscillation cycles.

When determining the period gradient in a growing tissue, the choice of reference frame and consideration of cellular movement is essential to define ROIs that follow cell trajectories. As we have not (yet) established simultaneous cell tracking and quantification of signaling oscillations in the same embryo using SPIM-for-4, we extracted the information on cellular motility from a reference dataset (McDole et al., 2018), where single cell tracking of E6.5-E8.5 mouse embryos was performed *in toto*. As SPIM-imaging of LuVeLu reporter embryos caused an altered axis elongation, likely due to phototoxic effects, the alignment to the reference tracking datasets needs careful consideration. We limited the comparison to a short time window (6 h) at the earliest stages of the imaging experiment, during which the growth of the mesoderm appeared qualitatively comparable in LuVeLu embryos and reference embryos (see Movies 12, 13). We assume that the qualitative characteristics of cell movement, i.e. mesoderm cells moving away from the wave origin, are preserved in the LuVeLu embryos. In addition, cell movement during this time window is approximately an order of magnitude slower than LuVeLu wave velocity (0.1-0.3 μm/min versus 3-10μm/min, see Fig. 5). Hence, any quantitative difference between LuVeLu and cell tracking datasets would, at the most, have a minor impact on our period measurements, and would not affect our conclusion that a period gradient exists at the onset of oscillations. Combined, our quantifications of wave onset dynamics thus provide a picture in which synchronous signaling activity is first established in a widespread mesoderm domain and, subsequently, a spatial period gradient causes the gradual onset of traveling wave patterns.

Several questions arise. First, how is the initial oscillation period gradient established? It is known that Wnt and Fgf signaling exhibit both graded and oscillatory activity during somitogenesis in mouse embryos (Aulehla et al., 2008; Dale et al., 2003; Dequéant et al., 2006), and as these pathways play a key functional role during gastrulation, it is of crucial importance to identify their signaling dynamics and functional link to the onset of segmentation clock oscillations described in this work. Optimized reporter lines enabling quantification of signaling dynamics at mouse gastrula stages are being developed and will be key to investigating how the period gradient is established and functionally controlled.

How the period gradient is functionally linked to the widespread pulse that we identified in mesodermal cells at the early bud stage is another key question that arises from our findings. So far, a functional role for the widespread pulse activity is lacking. One hypothesis is that the pulse may reflect an initial synchronization event that, when combined with the presence of an oscillation period gradient, inevitably leads into the emergence of coherent wave patterns.

A related question arises about the onset of the first synchronous activity per se. In principle, collective synchrony can be established via cues originating external to the ensemble of oscillating units. Alternatively, it can be based on the intrinsic ability of cells to couple and synchronize to their neighbors, i.e. rely on self-organization, without the need for external cue. In the first case, the ensemble can be labeled as being synchronous, while in the latter case, we would consider the cell ensemble to be synchronized. A plausible external trigger cue may, for example, be linked to the epithelial to mesenchymal transition (EMT) that cells undergo during gastrulation, as they ingress through the PS to form endoderm and mesoderm layers. As we found that *Lfng* expression initiates in the PS, timing of ingression may indeed constitute an external cue to individual nascent mesoderm cells. However, whereas mesoderm cells take about 1 day, from early streak to EB stage, to fully cover the egg cylinder (Kinder et al., 2001), our real-time quantifications revealed that the pre-clock pulse covers a widespread mesoderm domain in less than 5 h (Fig. 2G). Hence, at least a trigger with a constant delay from EMT to activation of *Lfng* pre-clock expression seems unlikely.

Alternatively, the observed collective synchrony established during gastrulation may reflect an internal, self-organized mechanism. Excitingly, recent *in vitro* studies have provided evidence for the potential of cultured PSM cells to exhibit signatures of collective synchronization of coupled oscillator ensembles (Tsiairis and Aulehla, 2017) but also for quorum sensing and switch-like behavior (Hubaud et al., 2017). A better understanding of similarities, and differences, between *in vivo* and *in vitro* models will be crucial to determine the significance of self-organization in the physiological *in vivo* context.

As a further extension of our work, we aim to combine the quantitative imaging approach with spatiotemporal functional control over signaling state, using, for example, entrainment (Sonnen et al., 2018) and optogenetic strategies, in order to gain a deeper understanding into the origin and function of collective signaling oscillations during embryonic patterning.

## MATERIALS AND METHODS

### Mice

The LfngT2A-3xVenus allele was generated with standard gene targeting techniques using R1 embryonic stem cells. The stop codon of the endogenous *Lfng* locus was targeted with a construct containing one selection cassette and two different reporter cassettes (3xVenus and 3xmCherry), a NLS and a PEST sequence. Each reporter cassette consisted of three times the fluorescent protein sequence, linked with a short peptide (GSAGS). FRT- and loxP sites flanked the reporter cassettes in a way that Cre-mediated recombination yielded the LfngT2A3xVenus allele in which the selection cassette and the 3xmCherry cassette were excised and the endogenous *Lfng* sequence is directly followed by a T2A sequence connected to the 3xVenus-NLS-PEST sequence (Fig. S3).

The mouse lines used in this study have been described previously: LuVeLu (Aulehla et al., 2008), R26-H2BmCherry (Abe et al., 2011; RIKEN CDB, CDB0239K), *Rbpj*^−/−^ (Han et al., 2002), Floxed RBP-J (Han et al., 2002; RIKEN RBR, RBRC01071), Hes7-null (Bessho et al., 2001; RIKEN RBR, RBRC05983), Hprt-Cre (Tang et al., 2002; Jackson Laboratory, 004302) and *Hes7*^−/−^ (Bessho et al., 2001).

All mouse lines were kept in an outbred background. Mice carrying the floxed RPB-J allele were first crossed to the Hprt-CRE deleter line (Tang et al., 2002; Jackson Laboratory, 004302) to generate *Rbpj*^wt/−^ animals, which were interbred to obtain *Rbpj*^−/−^ embryos. We dissected 122 embryos at 6.5 dpc and found that 19/30 *Rbpj*^−/−^ showed malformations and a developmental arrest that was not reported previously. In these litters, we found 1/24 *Rbpj*^wt/wt^ and 5/68 *Rbpj*^wt/−^ embryos that also showed impaired development at 6.5 dpc. We did not further investigate the underlying cause of this early phenotype but given that it appeared to be enriched within litters, we speculate that it could be due to additional environmental effects/stressors that manifest mainly in *Rbpj* embryos. For the analysis in this article, we used the *Rbpj*^−/−^ embryos (11/30) that showed no overt phenotype at 6.5 dpc, matching previous findings made with two different knockout alleles (Oka et al., 1995; Han et al., 2002). All animal experiments were conducted after project approval by the Institutional Animal Care and Use Committee (IACUC) and under veterinarian supervision, following the guidelines of the European Commission, Directive 2010/63/EU and AVMA Guidelines 2007.

### *In situ* hybridization

RNA *in situ* hybridization was performed as previously described (Wilkinson and Nieto, 1993) with the following published probe: *Lfng* (Aulehla and Johnson, 1999).

### *In situ* hybridization chain reaction

RNA *in situ* hybridization chain reaction (HCR) was performed as previously described (Choi et al., 2018) with adaptations for mouse embryo tissue as detailed by Sanchez et al. (2019). In addition, the modification of treating samples with 10 ug/ml proteinase K (Merck, CAS 38450-01-6) for 3 min at room temperature was made to prepare E8.5 whole-mouse embryos. Probe sequences used for *Msgn1*, *Uncx4.1* and *Shh* are provided in Table S1.

### Embryo dissection and mounting

Embryos were dissected in dissection medium [DMEM with 1 g l^−1^ glucose, no Phenol Red (Thermo Fisher, 11880028), 15% FCS, 2 mM L-glutamine, 1×penicillin-streptomycin and 20 mM HEPES] as described previously (Rivera-Pérez et al., 2010). Before mounting, dissected embryos were washed once in washing medium (dissection medium without HEPES, pre-equilibrated in 5% CO_2_). Embryos were transferred to a drop of mounting agarose (1.8% low melting temperature agarose in Leibowitz's L-15 medium, Thermo Fischer, 41300021) on a heating plate (42°C) and each embryo was embedded into a separate glass capillary (Brand, 701904) with a Teflon-tipped plunger (Brand, 701932). After allowing the agarose to harden for 5 min, the embryo was partially ejected, submerged in washing medium and agarose was removed with dissection forceps, leaving only the ectoplacental cone in agarose. Depending on the area of interest, embryos were mounted with the proximodistal axis either parallel (e.g. as in Fig. 1C) or perpendicular to the long axis of the capillary (Fig. 4A). For transport to the light-sheet microscope, embryos were pulled back into the capillary with some medium.

### Embryo culture system on the light-sheet microscope

The embryo culture chamber, chamber frame, SPIM-for-4 multi-sample holder and capillary caps were designed in SolidWorks (Dassault Systèmes) using CAD models of the Z.1 imaging chamber and the sample holder disc for syringes as geometrical references (kindly provided by Zeiss). The design of these parts is available from the Dryad Digital Repository (Falk et al., 2022): https://doi.org/10.5061/dryad.ht76hdrj2. All parts were manufactured in the European Molecular Biology Laboratory (EMBL) Mechanical Workshop. Culture chamber and capillary caps were made from medical grade plastic (PEEK; KTK).The SPIM-for-4 sample holder and culture chamber frame were made from aluminum.

The culture chamber had a 180° window towards the illumination and detection objectives and a window on the back of the chamber to monitor the sample during positioning with the camera mounted in the door of the Z.1 front system cavity. A 50 μm fluorinated ethylene propylene membrane (FEP; Katco) was glued around the three open sides of the culture chamber and a 11 mm round coverglass was glued into the window opening on the rear of the chamber using biocompatible silicone glue (Silpuran 4200; Wacker). FEP is routinely used as tubes (Kaufmann et al., 2012) or membranes (Strnad et al., 2016) to facilitate mounting in light-sheet microscopy because its refractive index matches that of water. The culture chamber was inserted into the chamber frame and both parts together were placed in the Z.1 imaging chamber (Fig. 1B). With a screw on the back of the chamber frame, the distance between front face of the culture chamber and the detection objective was adjusted to 0.5-1.0 mm.

An inlet and an outlet tube connected the culture chamber with the perfusion system. A 0.7×1.7 mm [inner diameter (ID)×outer diameter (OD)] silicon tube (Pro Liquid, 4001015HG_E) connected the chamber with a peristaltic tube pump outside the Z.1 front system cavity. From there, the medium was pumped into a gas equilibration chamber (Fig. S1) in which it was channeled through a coil of a 140 cm thin-walled 0.7×1.1 mm (ID×OD) silicon tube (Pro Liquid, 4001013HG). A thick-walled, less gas permeable 1.3×3.00 mm (ID×OD) tube (Tygon; Pro Liquid, 3700015) carried medium back into the culture chamber.

The equilibration chamber was made from a 100 ml blue cap bottle (Duran). It was heated to 38°C with heating films (Telemeter Electronic, HKAP2×2R5.4L12) glued around the bottle. Temperature was controlled with a temperature controller (Telemeter Electronic, TR12-G) and a Pt100 ceramic temperature probe (GHM-Greisinger, 602995) placed inside the equilibration chamber. The connecting tubes between equilibration chamber and embryo culture chamber were also heated by pumping warm water (40°C) through a spiral of tubing wrapped around them. The water was heated using the heating device Humidity S1 (Zeiss) that is usually used to preheat and humidify gas injected into the Z.1 sample chamber. The temperature sensor of the Z.1 sample chamber lies outside of the embryo culture chamber. To ensure stable 37°C inside the culture chamber, the Z.1 temperature control was set to 38.0-38.5°C (depending on the room temperature).

During experiments, the medium was circulated at 0.5 ml min^−1^. During preparation, pumping was accelerated for quicker medium equilibration. Generally, starting the perfusion system 10 min before introducing embryos was enough to stabilize the pH inside the culture chamber. The equilibration chamber was fed with a defined gas mixture using an in-house developed gas mixer. E6.5 dissected embryos were first cultured with 6% O_2_, 8% CO_2_ and 86% N_2_ then O_2_ was increased to 20% on day 7 while keeping CO_2_ at 8%. E7.5 dissected embryos were directly cultured with 20% O_2_. Higher O_2_ concentration increases the maturation rate of fluorescent proteins, resulting in a boost of fluorescent intensity and a better signal-to-noise ratio (Movie 4). To be able to detect the very first oscillations in LuVeLu embryos, O_2_ had to be increased early morning on day 7, before the occurrence of the oscillations (Fig. 2F). 8% CO_2_ was measured to produce the desired pH of 7.4 inside the culture chamber.

Embryos dissected at E6.5 were cultured in 75% rat serum and 25% DMEM (DMEM with 1 g L^−1^ glucose, no Phenol Red and 2 mM L-glutamine), supplemented with 1× penicillin-streptomycin. E7.5 dissected embryos were cultured in 50% rat serum, 50% DMEM, 1× penicillin-streptomycin. Before use in embryo culture, rat serum (male rats only; Envigo, S.R-0109D) was heat inactivated for 30 min at 56°C and centrifuged at 20,000 ***g*** for 1 h at 4°C to remove lipids that collect on the surface. The culture chamber and perfusion system was filled with 3.8 ml culture medium. 200 μl mineral oil (Sigma, M8410) and layered on top of the medium inside the culture chamber to reduce evaporation. For drug treatment experiments on the microscope, mineral oil was omitted to prevent that lipophilic drugs preferentially dissolved in the oil. Instead, wet tissues were placed underneath the imaging chamber to reduce evaporation. Control embryos shown in Fig. 3 and Fig. S6 consist of *Rbpj*^wt/wt^; LuVeLu^+/−^ and *Hes7*^wt/wt^; LuVeLu^+/−^ embryos, and therefore mineral oil was used during imaging.

Cleaning of the culture chamber with FEP membrane was routinely carried out in an ultrasound bath with dish detergent first, then 70% ethanol and finally double-distilled H_2_O, each treatment for 30 min. After 5-10 washes, the FEP membrane had to be replaced because of deteriorating optical properties. The perfusion system was flushed with water, 70% ethanol and water again after each experiment. Tubes were exchanged after drug experiments.

### Light-sheet imaging

All embryo live imaging experiments were carried out on the Light-sheet Z.1 (Carl Zeiss) using a 20×1.0NA Plan-Apochromat water immersion objective (Carl Zeiss), sequential illumination with both light sheets and 0.41× zoom. Images were taken with 1920×1920 pixel (0.5569 μm/pixel resolution in XY) at 16-bit. *Z*-stacks were generally recorded with 7.5 μm spacing (7.5 μm/pixel resolution in Z), 80-150 slices per sample, 300 ms exposure time and 10-20 min imaging interval. Imaging intervals and laser intensities were adjusted to the age of the embryos as younger embryos showed generally a higher susceptibility to photodamage. The described imaging settings resulted in datasets of 100-440 GB per embryo, depending on the number of channels and duration of the experiment, which was usually 20-40 h.

Venus/mVenus was routinely excited at 514 nm (50 mW internal laser power) and mCherry at 561 nm (20 mW internal laser power) or 514 nm if co-excited with Venus. Yellow and red fluorescence was detected with two cameras; the signal was split at 560 nm, narrowed with a 525/20 nm band-pass filter and a 585 nm longpass filter. For time-lapse experiments, LuVeLu^+/−^ embryos were excited at 2.0-2.4% laser power, R26-H2BmCherry^+/−^ embryos were excited at 1.2% laser power, LuVeLu^+/−^;R26-H2BmCherry^+/−^ double-positive embryos were excited jointly at 514 nm and 2.0-2.4% power, and fluorescence was imaged onto two cameras simultaneously. Some bleed-through of the Venus signal into the mCherry channel was evident but negligible, because the mCherry channel was not used for quantitative data analysis. For LfngT2A3xVenus^+/−^ live imaging, 2.8% laser power was used, snapshots of the *Lfng* expression domain at E6.5 were taken at 11.5% power and 120 ms exposure time, deviating from the standard settings described above. A full resolution snapshot of R26-H2BmCherry^+/-^ embryo imaging at E7.5 is available from the Dryad Digital Repository (Falk et al., 2022): https://doi.org/10.5061/dryad.ht76hdrj2.

### DAPT treatment

Embryos dissected at E6.5 [mid-streak (MS)/late streak (LS) stages] were first cultured in roller culture with 50 μM DAPT/culture medium (75% rat serum and 25% DMEM supplemented with 1× penicillin-streptomycin). After 12 h in roller culture, embryos were mounted onto capillaries and transferred to the light-sheet microscope. The embryos were imaged from early allantois bud stages onwards for 23+ hours in 50 μM DAPT/culture medium (50% rat serum and 50% DMEM supplemented with 1× penicillin-streptomycin).

### Time series registration

Throughout the text, 3D image dimensions will be used as depicted in Fig. 1A: X, horizontal (perpendicular to the detection light path); Y, vertical; Z, horizontal (parallel to the detection light path). Two images were acquired per image stack position with the two opposing light-sheets. During acquisition, both images were combined using a mean fusion algorithm provided with the microscope control and image processing software ZEN (Carl Zeiss). For further processing, datasets were subsampled by a factor of two or three in X and Y also in ZEN.

Rigid image registration (translation and rotation) of 3D+time (3D+t) datasets was carried out using an in-house developed ImageJ/Fiji (Schindelin et al., 2012) script (available on https://github.com/tischi/fiji-script-registrationUsingElastix), providing a graphical user interface to the command line registration tool elastix (Klein et al., 2010; Shamonin et al., 2013). Registration was performed in a recursive manner: An initial reference time point was defined by the user. The transformation from the successive frame Fn+1 to the reference frame Fn was calculated and applied to Fn+1. The resulting transformed frame served as a new reference Fn for the transformation of the next Fn+1, and so on. If the initial reference frame was not the first frame, the procedure was also propagated in the Fn-x direction. Loading and browsing of the large datasets before and after registration was carried out with an in-house developed ImageJ plugin for streaming of Tiff and HDF5-based image stacks (available on https://github.com/rmd13/fiji-plugin-bigDataTools).

After registration, deviations of the proximodistal and anterioposterior axes of the embryo from the Y and Z image axes, respectively, were adjusted, using a custom-made ImageJ macro: reorientation parameters were determined manually for a user-defined reference frame and subsequently applied globally to all time points. To cope with the size of datasets, time points were loaded and processed sequentially.

### Landmark segmentation

Owing to the drastic size and shape changes of the growing mouse embryo, morphological landmarks like the allantois or the node change their absolute position within the 3D+t datasets over time even after automatic registration. An additional step of landmark segmentation was performed to be able to follow relative positions in the embryo over time. The spatial extent of the LuVeLu-positive domain was manually marked with a rectangular bounding box in several reference frames of projections along Y and Z throughout the time series, and the position and size change of the bounding box was linearly interpolated for the intermediate time points. This information was used to make intensity profiles and kymographs relative to the size of the LuVeLu expression domain.

### Transverse optical sections

Transverse sections (XY planes) of embryo 3D+t datasets were generated in Fiji. Sections were positioned at 50% of the proximodistal extent of the manually segmented LuVeLu domain for each time point (Fig. 2D). Each transverse section image represents a maximum intensity projection (MIP) of a 12 μm slice through the embryo.

### Kymographs and intensity profiles from maximum intensity projections

Kymographs along the propagation direction of waves were generated in Fiji from spline curves in MIPs (projected along Y or Z) of 3D+t embryo datasets. For each time point, the spline curve was scaled and positioned relative to the manually segmented LuVeLu domain as described above (Landmark segmentation). MIPs were smoothed with a 2D Gaussian (=12 μm) and intensity was averaged in a 25 μm corridor along the spline curve.

For line profiles in kymographs, intensities in a 10 pixel (≈14 μm) wide stripe were averaged. For normalized plots in Fig. 2G,H, profiles were processed with locally weighted scatter plot smoothing (LOWESS) in Matlab (Mathworks; bandwidth α=0.06), background was subtracted and data were normalized to the maximum value. For plots in Fig. 3B,C, raw intensity profiles obtained from the kymographs were plotted without smoothing or normalization.

### Surface kymographs

For kymographs analyzed in Fig. 5, the curved surface of the mesoderm was followed along the propagation direction of waves with the following strategy. First, 3D+t datasets were acquired by imaging E7.5 LuVeLu^+/−^ embryos from the distal end for ∼24 h (*n*=5). Images were taken with 1920×1920 pixel in XY (0.5569 μm/pixel resolution in XY), Z-stacks were recorded with 7.5 μm spacing (7.5 μm/pixel resolution in Z), 150 slices per sample, with 10 min imaging interval. These datasets were downsized by subsampling with a factor of 3.75 in X and Y. Subsequently, these datasets were processed with temporal background reduction. In this procedure, a fixed 30×30×150 voxel in the data outside the embryo was averaged to obtain the background value for each time frame, then subtracted from the entire image. An offset value of 1000 intensity counts were added subsequently to the image uniformly. These images were then registered as described in ‘Time series registration’. After registration, the datasets were resized in the Z dimension by a factor of 3.59 to obtain isotropic resolution in X, Y and Z (2.088 μm/pixel resolution in XYZ). The datasets were then thresholded with a value of 1030 to make binary masks for each timepoint. These masks were summed for all timepoints, and its center of mass (COM) was calculated to be used as the center coordinate of the embryo. The entire 3D+t dataset was translated so that the COM coordinate matched the center of the image, then rotated with manually defined angles around the COM such that the anterioposterior and proximodistal axes matched the Y and Z axes, respectively.

3D coordinates of the proximal oscillation origin and distal ends of the PSM on both sides were manually marked throughout the time series in several key frames, then linearly interpolated for the intermediate timepoints. For each timepoint, the midpoint between the origin and a distal end were calculated. From the image, line profiles extending the vector from the COM to the midpoints were obtained, and the coordinate with the maximum intensity in this line profile was recorded for all timepoints. A spline was fitted to these coordinates through time, and its values were used to designate the coordinate of an intermediate point on the mesoderm surface between the oscillation origin and the distal end of the PSM for each timepoint.

A spline was fitted through these three points (proximal oscillation origin, intermediate point and distal end) at each timepoint to be used as the line of interest (Movie 12). To calculate the distance of the arc between the proximal oscillation origin and the distal end, we took a chorded approximation approach by dividing the arc into 100,000 piecewise linear segments with equal division of the spline parameter u=[0,1]. The cumulative sum of these segment lengths were stored in a table with the corresponding parameter value, and the total sum was stored as an estimate of the total arc length. The u value giving the closest cumulative distance to each integer distance was determined from the table for all integers below the total arc length. Points on the arc with these u values were used to collect values for the kymograph, so that one spatial pixel on the kymograph corresponds to one pixel distance on the arc. To collect intensity values, the 3D dataset was smoothed with a 3D Gaussian (σ=6 pixels ≈12.5 μm) and intensity was averaged within a spherical volume centered at each point with a diameter of 12 pixels ≈25 μm. The kymograph was constructed by fixing the intensity time series from the origin at the midline and extending the values collected from the left and right arcs on top and bottom of the midline, respectively.

All computational steps described here for surface kymograph generation were implemented in the Python programming language, making extensive use of the ‘SciPy’ library (Jones et al., 2001) and ‘OpenCV’ library (Bradski, 2000).

### Phase kymographs

For each surface kymograph, the timepoint at which the waves began after the LuVeLu ‘pulse’ was determined manually. The preceding frames were cropped out for the subsequent phase extraction. Regions outside the kymograph (regions exceeding the total spline length at each timepoint) were filled with a background value of 1067. The intensity kymographs were smoothed with a Gaussian in the spatial dimension (σ=5 pixels ≈10.4 μm).

Phase extraction from the raw intensity kymographs was carried out using Wavelet transforms (Torrence et al., 1998). To remove low-frequency trends, every row (in the following called time-series) of a kymograph was convolved with a Sinc filter with a ‘cut-off-period’ of 220 min. Sinc filters are designed to be optimal low-pass filters in the frequency domain (Smith, 1997). The results from the convolution were then subtracted from the original time series. These detrended time series were then convolved with 600 complex Morlet Wavelets, scanning periods from 100 to 220 min. By tracing the power-maxima in the Wavelet spectra over time, the so called ‘ridges’ were identified. Evaluating the complex Wavelet transforms along the ridges gave a complex signal for every time series. Finally, by extracting the argument from these complex signals, the phase values over time were obtained. Re-stacking these phase-valued time series produced the phase kymographs (Fig. S8B). The module of wavelet transform for time series used in this study is available as pyBOAT (Mönke et al., 2020 preprint) (available at https://github.com/tensionhead/pyBOAT).

### Cell movement extraction from *in toto* cell tracking datasets

To extract cell movement from the *in toto* cell tracking datasets presented by McDole et al. (2018), we first temporally aligned each of the four tracking datasets in relation to the LuVeLu imaged embryos by designating a timepoint corresponding to the peak of the ‘pulse’ stage. This timepoint was manually determined by comparing the extent of mesoderm ingression and the diameter of the embryo between the tracking and LuVeLu datasets. The start of waves (t=0) was designated to be 260 min after the peak of the ‘pulse’ for each embryo. For spatial registration of the datasets, we first defined the embryo midline for all time points using landmark annotations in the cell tracking datasets. In the tracking datasets, we set the location of the LuVeLu wave origin using an estimation based on the designated cell fates as input. The lines of interest (LOI) used to quantify oscillations in the LuVeLu datasets were used to localize the corresponding positions in the tracking datasets.

These projected LOI were used to select mesoderm cell tracks in each tracking dataset. Cell tracks that remained within 30 μm of the LOI for over 300 consecutive minutes were included.

Using the position of wave origin and t=0 as a common reference, the acquired trajectories were averaged into vector fields, either with all four datasets (Fig. 5C) or individually (Fig. S8A). The final streamline plot was generated from the vector field using a built-in function in the ‘Matplotlib’ library.

All computational steps described here for extracting the cell movement data were implemented in the Python programming language. Visualizations (Movie 13) were carried out using Blender (available on http://www.blender.org).

### Periods

For all measurements made for periods, only the left side of the embryo, or the top half of the kymograph, was used, to match the number of proximal and distal samples (*n*=5).

To calculate the distal periods for solid lines in Fig. 5G, the intensity values along the contour paths of the boundaries of the intensity kymograph were taken as input time series, as these paths moved proportionally to the expansion of the mesoderm area. To ensure the contour paths were inside the oscillatory domain, they were shifted from the boundaries of the kymograph towards the midline by 20 pixels ≈41.8 μm. The intensities along the midline were taken as the proximal time series. Intensity was averaged in an 8 pixel ≈16.7 μm wide corridor along these paths. These time series were subsequently analyzed by Wavelets as described in ‘Phase kymographs’. The periods were extracted along the maxima ridges of the Wavelet spectra. Period values during 2∼6 h after the initiation of waves were collected as data points (24 points per embryo per region), which were used to calculate median and IQR for Fig. 5G.

### Phase gradient slope and wave number q

For all measurements made for wave number, only the left side of the embryo, or the top half of the kymograph, was used, to correspond to period measurements (*n*=5).

The phases along a column of the phase kymographs were first unwrapped to obtain continuous phase values. At each column, 20 pixels at the distal end were cropped off to match period measurements (see ‘Phase kymographs’). The phase difference between the distal end and midline were taken and divided by 2π to obtain wave numbers (Fig. 5E). The length of the cropped column, corresponding to spline length on the surface of the mesoderm, was used as mesoderm length (Fig. 5D). The wave number was divided by the mesoderm length at each timepoint to obtain the linear estimate of the phase gradient slope (Fig. 5F).

### Implications of projection-based analysis on the obtained measurements

Measuring signaling dynamics inside the intact, growing embryo poses certain geometrical challenges. As the paraxial mesoderm during gastrulation may be described as a thin sheet curved around the cup-shaped epiblast, measurements along fixed projections, as we used them as basis for our analysis, naturally introduce spatial distortions. However, the use of kymographs obtained by fixed projections have been limited to mainly make qualitative conclusions such as describing the existence of the ‘pulse’ and wave dynamics. We note that our most prominent quantitative read-outs, wave numbers (Fig. 5E), phase gradient slope (Fig. 5F) and period measurements (Fig. 5G), have been carried out on surface kymographs to avoid these spatial distortions.

We therefore conclude that the benefits of our approach, namely the substantial reduction of data volume (∼100-fold) by using fixed projections of three-dimensional image data for analysis, outweighed the aforementioned inaccuracy in the spatial dimension, especially when only qualitative description was necessary.

### Selection criteria for sample sizes

Quality of embryo development on the microscope was subject to a certain variability that may have been impacted by the quality of the embryo starting material, as well as irregularities in the dissection, mounting and culture procedure. We primarily used morphological features to judge the quality of development (size, axis definition, head fold and neural tube) and only included embryos in the analysis that were similarly developed as *in utero* developed embryos of a corresponding age. For embryos that were not DAPT treated, we also included the presence of sustained waves of LuVeLu/LfngT2A3xVenus signaling reporter as criterion to include in any analysis.

For the assessment of culture outcome (Fig. 1E, Fig. S2), no selection of embryos was made. Six out of six embryos subjected to SPIM-imaging of LuVeLu reporter, 4/4 embryos subjected to SPIM-imaging of the nuclear marker H2B-mCherry, 9/10 embryos cultured on a microscope without imaging and 29/30 embryos cultured on roller culture over five independent imaging experiments were considered for Fig. S2. One embryo from the group cultured on a microscope without imaging was lost during the RNA *in situ* hybridization chain reaction (HCR) process, hence the 9/10 usage of samples in this group. One embryo from the group cultured on roller culture was deformed during the mounting process, hence the 29/30 usage of samples in this group.

For the quantitative analyses made for Fig. 5, a total of eight embryos carrying the LuVeLu reporter were imaged in two independent experiments. Of these eight embryos, five embryos were used for the final analysis. Of the three embryos omitted from the analysis, one embryo showed weak LuVeLu expression and no visible oscillations. The remaining two omitted embryos showed visible LuVeLu oscillations; however, they either moved out of the field of view during imaging or were not imaged long enough to capture 800+ min of oscillation dynamics and were therefore omitted from analysis. No selection of embryos based on sex were made throughout this study.

## Supplementary Material

Supplementary information
