# Supplementary material for: Imaging the onset of oscillatory signaling dynamics during mouse embryo gastrulation
Source: Development. Author manuscript; Available in PMC 2022 Aug 13. (PMC9340547; doi:10.1242/dev.200083)
Supplement: Supplementary Information [file EMS151782-supplement-Supplementary_Information.pdf]

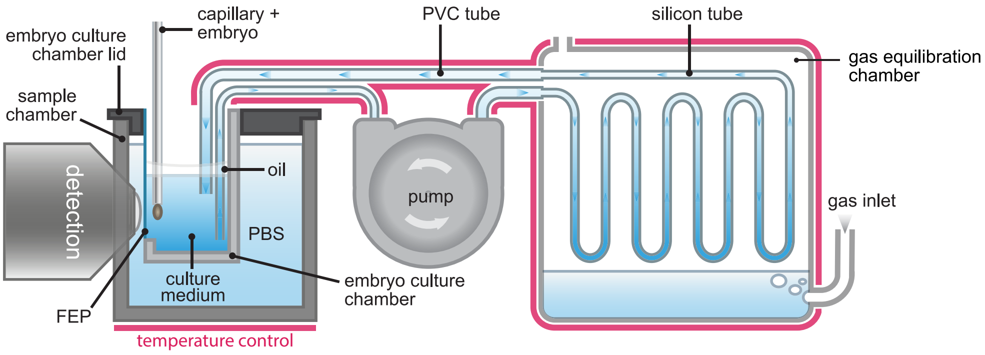

Fig. S1. Medium perfusion system for live embryo imaging on SPIM. Schematic section view through the Z.1 sample chamber with inserted embryo culture chamber and attached closed-cycle perfusion system. The embryo culture chamber sits inside the Z.1 sample chamber which is filled with PBS. Only the embryo culture chamber and the perfusion system are filled with culture medium. The medium in the culture chamber is covered with mineral oil to reduce evaporation. Medium is continuously pumped through the chamber and the perfusion system that is positioned outside of the front system cavity of the microscope. In the gas equilibration chamber the medium is saturated with a defined gas mixture before it is reintroduced into the culture chamber. Thin-walled, gas permeable silicon tubes are used inside the equilibration chamber, thick PVC tubes are used outside. The entire system is heated (indicated in red).

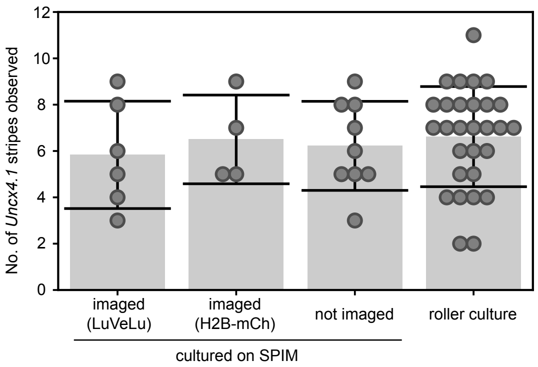

Fig. S2. Comparison of culture outcome on SPIM and roller culture Bar plot showing the number of Uncx4.1 stripes, indicative of segments formed during following experimental conditions: SPIM-imaging of LuVeLu reporter (mean:5.83, n=6), SPIM-imaging of nuclear marker H2B-mCherry (mean:6.5, n=4), cultured on the microscope but not imaged (mean:6.2, n=9), and cultured in roller culture (mean:6.6, n=29). Embryos were dissected at E7.5 and cultured for 24 hours. Bar plot shows mean value of samples within each group, error bars show standard deviation.

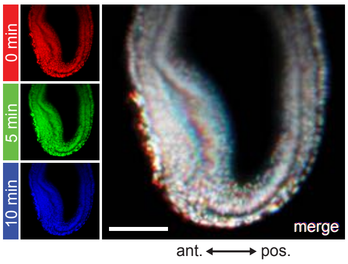

Fig. S3. Image stability test with multi sample imaging. An E7.5 mouse embryo expressing R26-H2BmCherry was imaged in a multi-sample imaging routine. Three consecutive time-frames of the same imaging location are shown (left). In between these frames, three other embryos were imaged (not shown). Frames were color coded in red, green, and blue and overlaid (right) to visualize positional stability. Pixels with the same intensity in all three images appear white. The visible shift is due to morphological changes of the embryo. Scale bar: 100 µm.

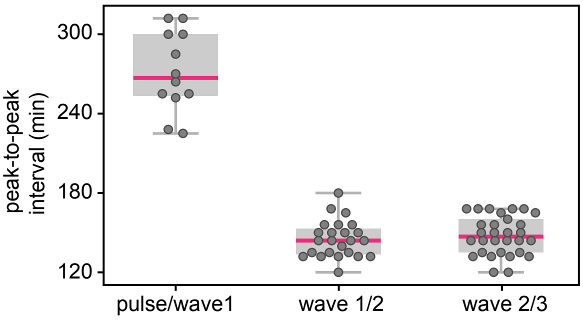

Fig. S4. Timing of pulse and the first waves. Boxplot showing the peak-to-peak intervals between the pulse and the first three waves measured in profiles as shown in Fig. 2G for different embryos. Time intervals (given as median with IQR) from pulse peak to wave 1: 267 min (46.5 min, n=12), from wave 1 to wave 2: 144 min (17.7 min, n=24), from wave 2 to wave 3: 147 min (25.2 min, n=30). Sample sizes differ between measurements because the start of the imaging experiment varied in respect to the developmental stage of the embryos. Consequently, some embryos were already too far developed to capture e.g. the pulse.

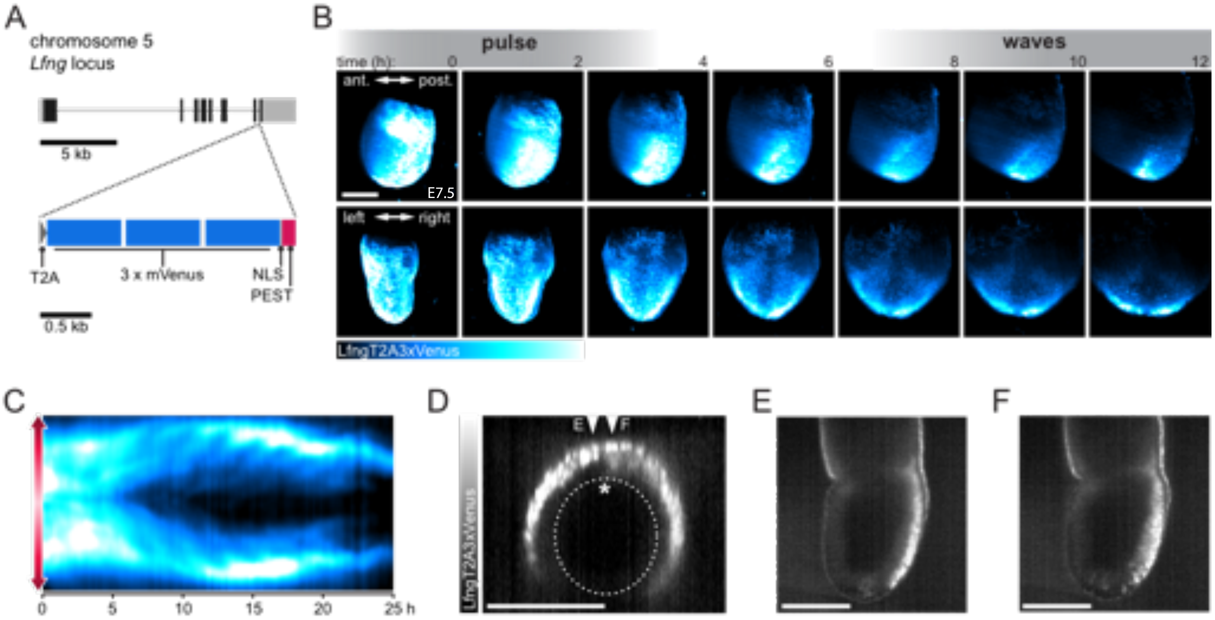

Fig. S5. *Lfng* expression dynamics during gastrulation, monitored using LfngT2A3xVenus knock-in reporter. (A) Schematic representation of the LfngT2A3xVenus construct knocked into the *Lfng* locus at the C-terminus (NLS, nuclear localization signal). (B) Still frames of a LfngT2A3xVenus embryo imaged from mid-gastrulation onwards. Maximum intensity projections in side view (top; ant., anterior; post., posterior) and from posterior (bottom) are shown. In the first two frames the mVenus signal is partially obscured by strong autofluorescence of the visceral endoderm. (C) Kymograph along the direction of wave propagation as illustrated in Fig. 2E for the embryo shown in (B). (D) Transverse section through a late streak embryo showing *Lfng* reporter fluorescence in mesoderm and PS (asterisk; amniotic cavity is outlined for orientation). (E,F) Sagittal sections of the same dataset as in (D) lateral to the PS (E) and at the PS (F) showing that *Lfng* reporter expression in epiblast is restricted to the PS. All scale bars: 200 µm.

Development • Supplementary information

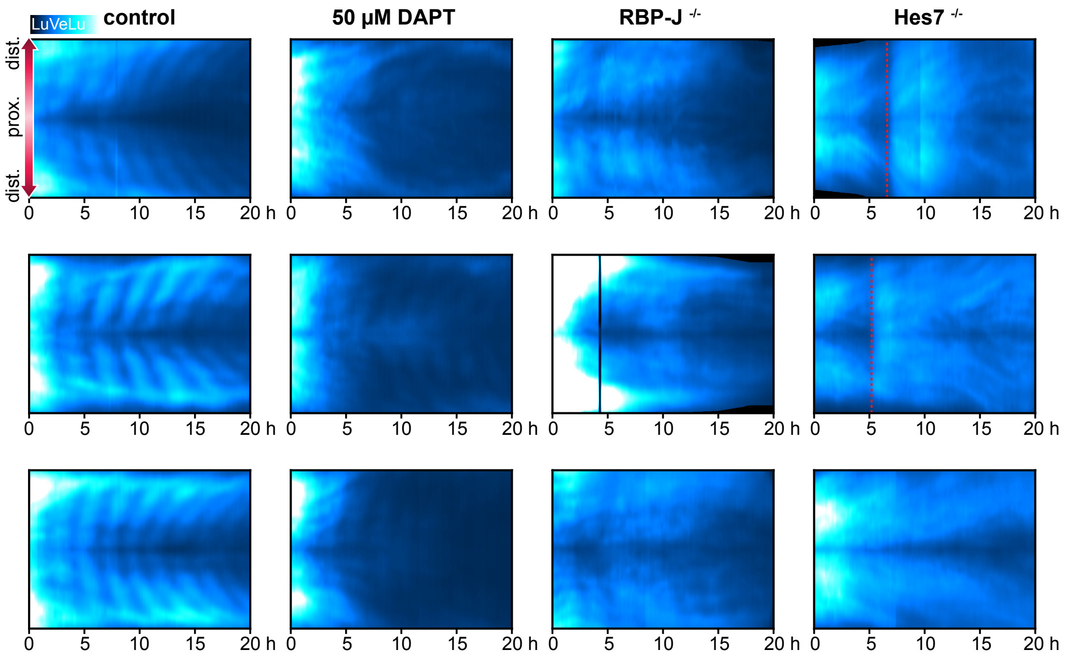

Fig. S6. Additional *LuVeLu* kymographs of DAPT treated, *RBPjk* knockout, and *Hes7* knockout embryos. In relation to Figure 3. Three additional samples of LuVeLu fluorescence intensity kymographs are shown for each condition (control, 50µM DAPT, *RBPjk*$^{-/-}$,*Hes7*$^{-/-}$). Red dashed lines in the two kymographs for the *Hes7*$^{-/-}$ samples indicate the timing when O_2 concentration was increased from 6% to 20%, explaining the abrupt rise in fluorescent intensity. Brightness and contrast were set identically for all samples.

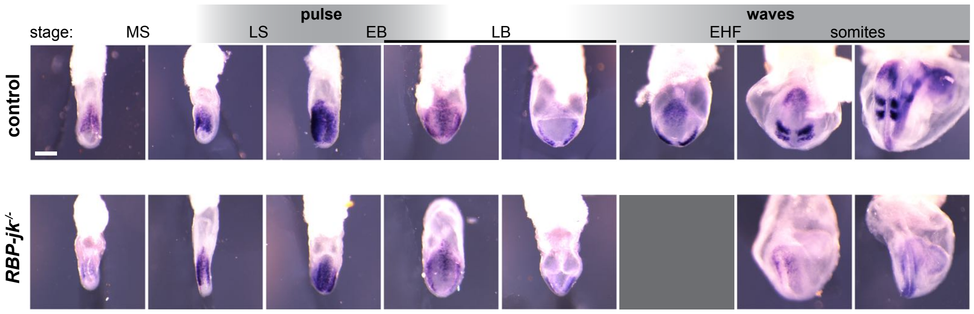

Fig. S7. *Lfng* expression in control and *RBPjk* knockout embryos. in situ mRNA hybridization was performed for *Lfng* in control and *RBPjk* knockout embryos. Control *RBPjk*$^{+/+}$, *RBPjk*$^{+/-}$ (top row), and knockout *RBPjk*$^{-/-}$ (bottom row) embryos came from the same litters and were stained together. Number of control/*RBPjk*$^{-/-}$ samples collected for the different stages: MS: 1/3, LS: 6/3, EB: 9/2, LB(1): 12/3, LB(2): 11/1, EHF: 16/0, somites(1): 6/6, somites(2): 8/1. Scale bar: 200 µm.

Development • Supplementary information

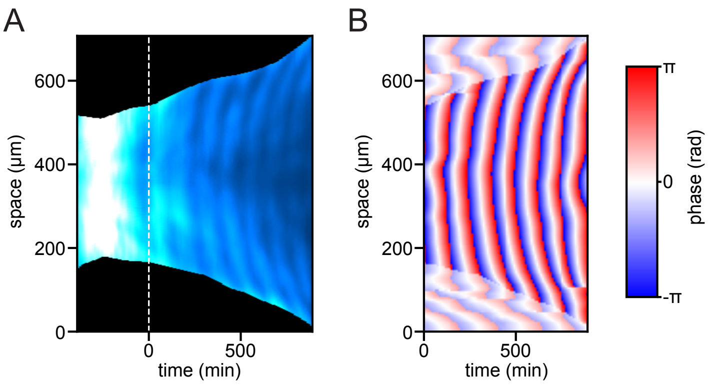

Fig. S8. Generation of phase kymographs from intensity kymographs (A) Representative LuVeLu$^{+/-}$ intensity kymograph made by following the surface of the mesoderm from Fig. 5A. Regions outside the kymograph was filled with a background value of 1067 prior to wavelet analysis. White dashed line represents a manually determined timepoint zero, the start of wave dynamics. The timeframes before timepoint zero were cropped prior to generating phase kymographs. (B) Correponding phase kymograph made by analyzing intensity time series in (A) with Wavelets. Shaded region represents region outside of the kymograph, corresponding to black regions in (A).

Development • Supplementary information

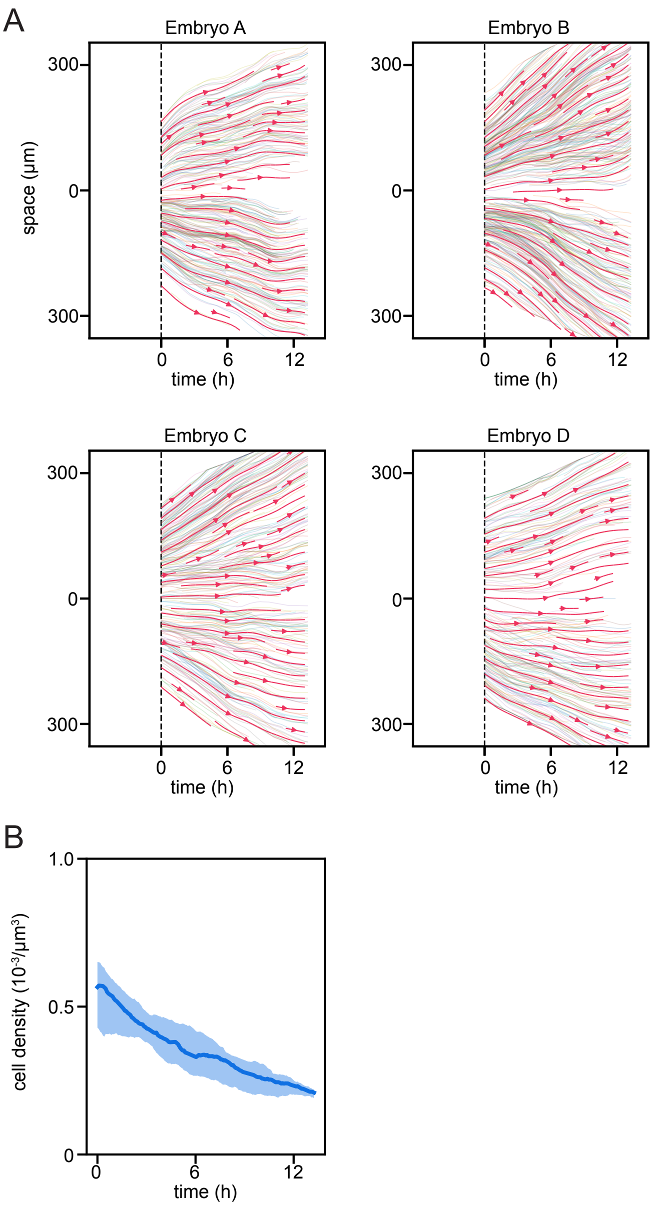

Fig. S9. Cell track and density measurements from cell tracking datasets (A) Streamline plot showing flow of cells, based on cell tracks obtained from four mouse embryo datasets, shown individ-ually. Cell tracks were obtained by transferring the line of interest used to generate surface kymographs. The proximal region is registered at the center, while the left and right distal ends extend above and below, respectively. Each shaded line represents a single cell track. Annotated mesoderm cells staying within the 30µm radius of the line of interest for more than 300 min were included for analysis. 1/2 of the set of cell tracks are visualized per embryo. (B) Cell density measured in the volume within the 30µm radius of the line of interest on the cell tracking datasets. All annotated mesoderm cells in this zone were considered for each timepoint, for left and right sides of the embryo independently. Solid lines represent median, corridors mark IQR. (A–B) Time is registered to 0hr corresponding to the start of the first wave in LuVeLu embryos.

Table S1. Sequences of probes used for *In situ* hybridization chain reaction (HCR) Following (Choi et al. 2018), pairs of split-initiator probes were designed for each gene. Five pairs were used for *Msgn1* and *Shh*, and ten pairs were used for *Uncx4.1*. Uppercase letters represent hybridization sequences and lowercase letters represent split initiator sequences.

target gene	ID	sequence
Msgn1	Even 01	ctcactcccaatctctataaTCCTCCAGGCTGAGGAAGGTCTCAC
	Odd 01	CCAGCGGTGTCAGAAGAGTCCAGGCaactaccctacaaatccaat
	Even 02	ctcactcccaatctctataaGAAGGAGCTGGGGAGAGGCTTTGAG
	Odd 02	AGTGCGACCTCAGAGTAGGACTCCAaactaccctacaaatccaat
	Even 03	ctcactcccaatctctataaAGCCAACATGCTGTAATCCAGCTCG
	Odd 03	AGCAGTGTGTAGATAGGGAGGTTGAaactaccctacaaatccaat
	Even 04	ctcactcccaatctctataaAGTTTCTCTCTCTCGCTGGCCTTCC
	Odd 04	AGGGCATCGGCTAAGGTCCGCATCCaactaccctacaaatccaat
	Even 05	ctcactcccaatctctataaTGTACTTGAGTGTCTGGATCTTGGT
	Odd 05	CTGTGAGTTCCCCGATGTACTTGATaactaccctacaaatccaat
Shh	Even 01	cctcgtaaatcctcatcaaaACTTGTGAGCTGTCCCCGCGCTGTC
	Odd 01	GCAGCATCTCGTCCGCGGAACCTGAaaatcatccagtaaaccgcc
	Even 02	cctcgtaaatcctcatcaaaTACTTGCTGCGGTCCCGGTCGGACG
	Odd 02	TCCACAGCCAGGCGAGCCAGCATGCaaatcatccagtaaaccgcc
	Even 03	cctcgtaaatcctcatcaaaGAGAATGGTGCCGTGCGCCGTGAGC
	Odd 03	GTAGCACGAGGCGAGCACCCGGTTGaaatcatccagtaaaccgcc
	Even 04	cctcgtaaatcctcatcaaaCCTTATAGTCTACTTTGGACTGCCC
	Odd 04	CAACAGAACTCCCCGGGGTTTTTGCaaatcatccagtaaaccgcc
	Even 05	cctcgtaaatcctcatcaaaCAAACCAAGAAGGCAGTACAGAAGA
	Odd 05	GACAAAGTGGCGGTTACAAAGCAAAaaatcatccagtaaaccgcc
Uncx4.1	Odd 01	cctcaacctacctccaacaaTGAAGGGCTGGCTTTCGCCGGCCAC
	Even 01	TGTCTGGATCCCCCGAGTCTGCAAGattctcaccatattcgcttc
	Odd 02	cctcaacctacctccaacaaTGCCAGCCGGTAAAGTTGGTGCGGG
	Even 02	TTGAACGCCTTCTCCAGCTCCTCCAattctcaccatattcgcttc
	Odd 03	cctcaacctacctccaacaaGATTTTGGAACCAGACCTGAACTCG
	Even 03	TCTCCTTCTTTCTCCATTTGGCCCGattctcaccatattcgcttc
	Odd 04	cctcaacctacctccaacaaGAGCAGTTTCTTCTCGTGTTTGCGT
	Even 04	CGAGTGCAGGTGGCGGCTCTGACTCattctcaccatattcgcttc
	Odd 05	cctcaacctacctccaacaaTAGCCTTAGGCAGGCCCGCAGTCGC
	Even 05	ACAGCAGGCTCTCCACGCTGAACGGattctcaccatattcgcttc
	Odd 06	cctcaacctacctccaacaaCCAATCAGGGTCCGAGGTGCACACG
	Even 06	ATGGGGTAGAGCAAGAAGTGGCCCTattctcaccatattcgcttc
	Odd 07	cctcaacctacctccaacaaTGGCCCGGGAGAGGCAACAACCTCA
	Even 07	TAGGTCCCGGAAAGAAGCGGGAGCGattctcaccatattcgcttc
	Odd 08	cctcaacctacctccaacaaGTCCATGTCCACCTCCTCGCCCTCG
	Even 08	CATCTTCCAGCCGCGGTCCCCAGCTattctcaccatattcgcttc
	Odd 09	cctcaacctacctccaacaaACCTCACCCCAGGACCAGCAGCTGC
	Even 09	GGTTTTTTGATCCCTCCGGAGGAGGattctcaccatattcgcttc
	Odd 10	cctcaacctacctccaacaaTGTGGGTTTTGTGTGTGTGTGTGTG
	Even 10	GCATTTTACAAGGCTGAGGATTCTCattctcaccatattcgcttc

Development • Supplementary information

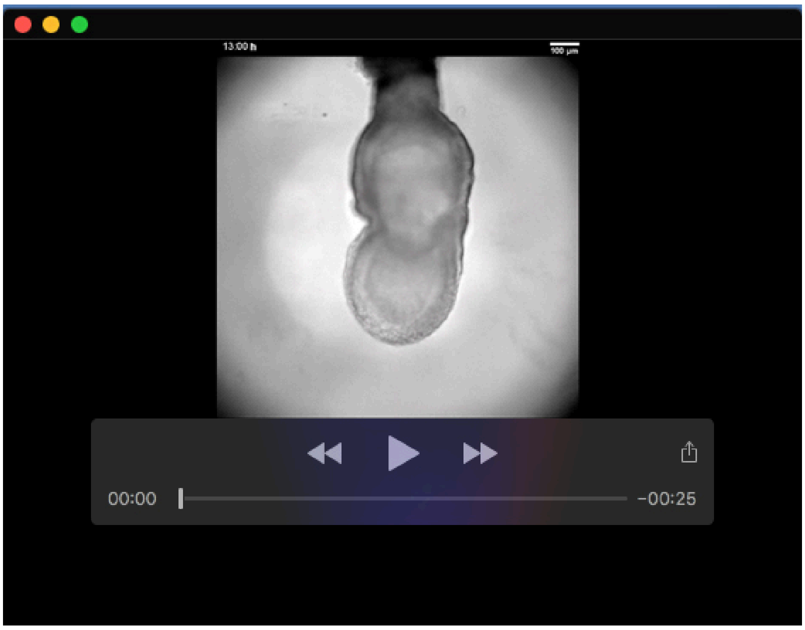

Movie 1. 40 h development of a mouse embryo from early gastrulation to somitogenesis. Related to Fig. 1D. The movie shows the bright-field signal of an embryo developing on the Z.1 light-sheet microscope using the customized culture and mounting setup. The field of view is readjusted at 16:20 h to account for the embryo growth. The embryo was imaged every 20 min but is shown at ˜1 h intervals because acquisition failed for a number of frames due to a software problem of the microscope. Time is indicated in hr:min.

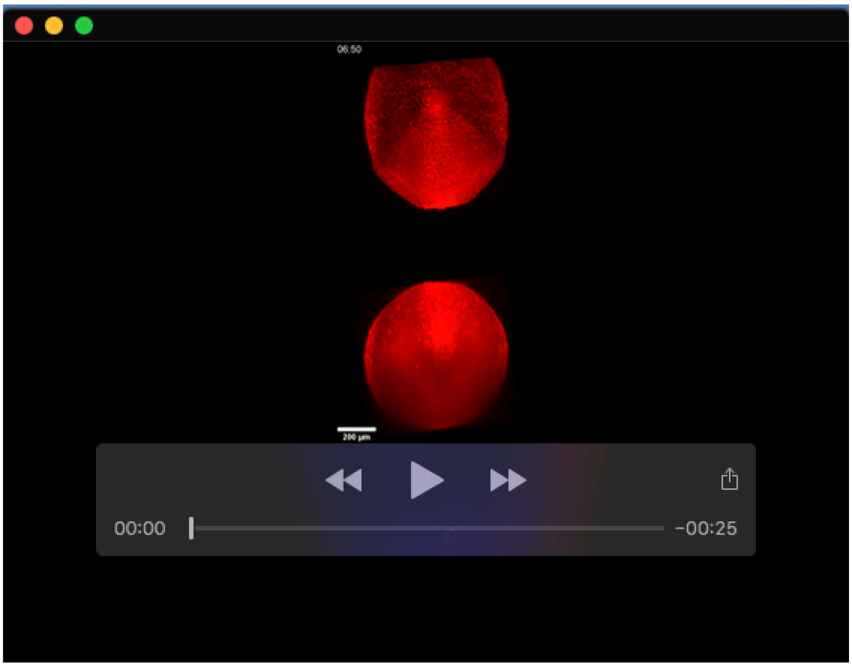

Movie 2. Imaging of R26-H2BmCherry$^{+/-}$ embryos prior to RNA *in situ* hybridization chain reaction (HCR) Related to Fig. 1E(ii). An embryo expressing the nuclear marker H2BmCherry (red) was light-sheet imaged from E7.5 for 24 hrs, with Z-stacks with 7.5 µm spacing, 120 slices per sample, 200 ms exposure time with 1.2% 20mW 561nm laser and 10 min imaging interval. The movie shows the embryos as MIP from posterior (top) and distal orientation (bottom). Time is indicated in hr:min.

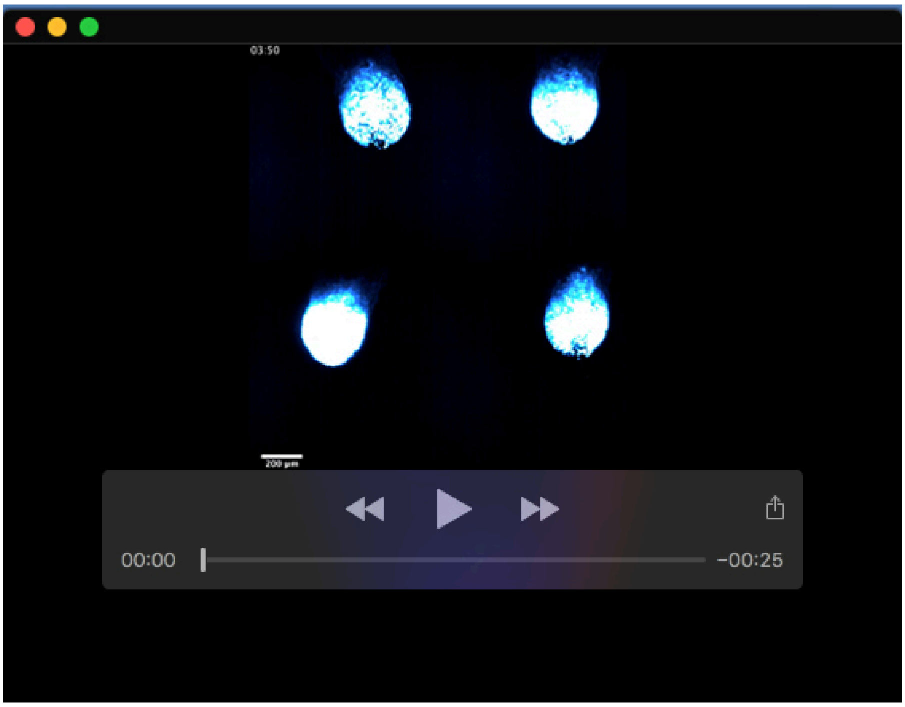

Movie 3. Four embryos imaged simultaneously in a single experiment using SPIM-for-4. Related to Fig. 1F,G. Four embryos expressing the dynamic Notch signaling reporter LuVeLu (cyan) was simultaneously light-sheet imaged from E7.5 for 28 hrs using SPIM-for-4. The movie shows the embryos as MIP from posterior view. Time is indicated in hr:min.

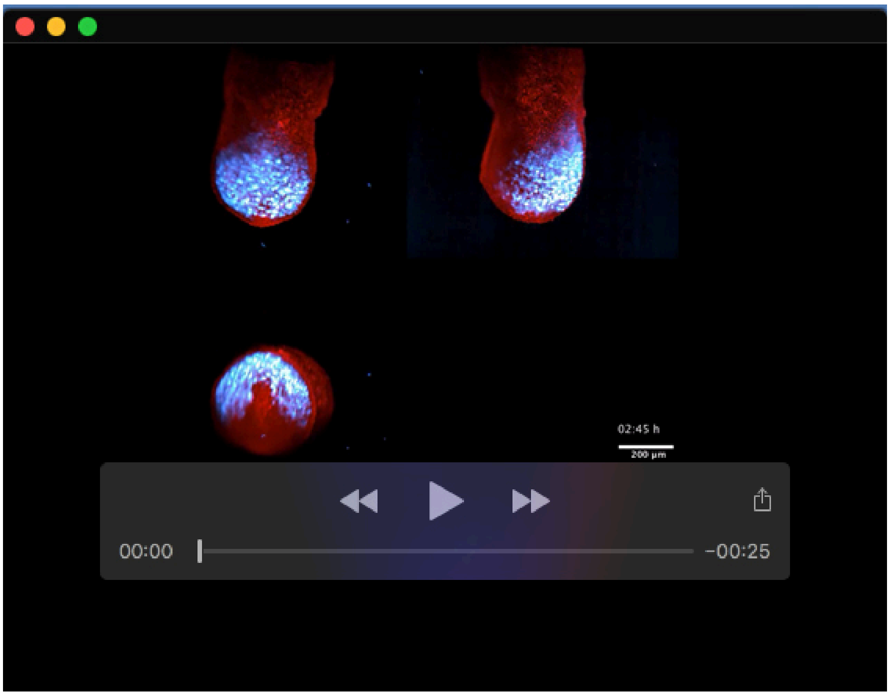

Movie 4. Dynamic activity patterns of LuVeLu before and during early somitogenesis. Related to Fig. 2A. An embryo expressing dynamic Notch signaling reporter LuVeLu (cyan) and nucleus marker R26-H2BmCherry (red) was light-sheet imaged from E6.5 to E8.5. The movie shows the embryo as MIP from three perspectives: posterior (top left), left side (top right), distal (bottom left). Posterior and side view were recorded as separate image stacks from different angles, the distal view is generated from the same dataset as the posterior view, projected along the proximodistal embryo axis. Note the sudden increase of LuVeLu signal at 12:30 h which is caused by the switch from 6% to 20% O_2 in the embryo culture environment (see Materials and methods). Time is indicated in hr:min.

Development • Supplementary information

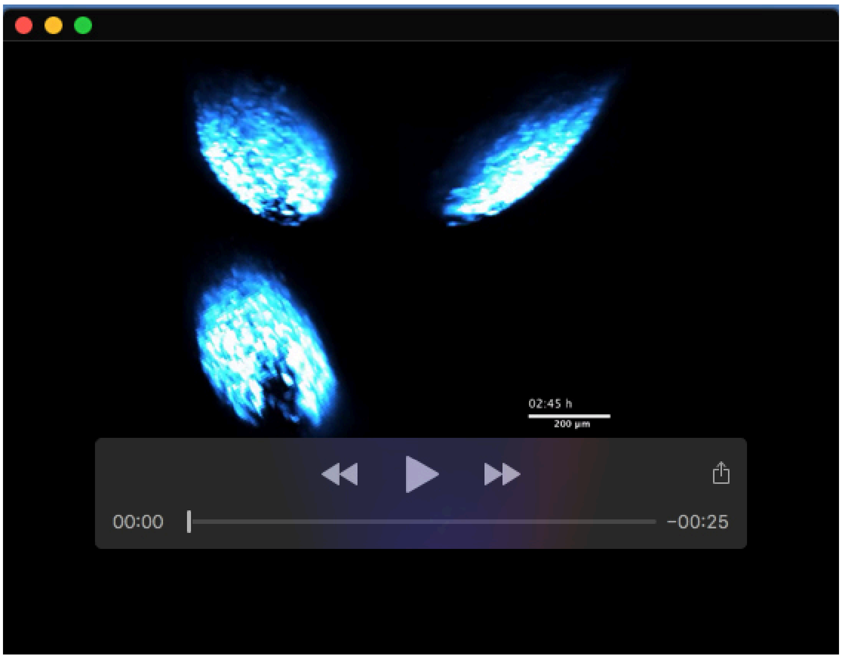

Movie 5. Visualizing the pulse and the very first LuVeLu waves. Related to Fig. 2F. The movie shows LuVeLu fluorescence in an embryo imaged from LS stage onwards from posterior side. The embryo is shown as MIP from three perspectives: posterior (top left), left side (top right), distal (bottom left). The distortion of the embryo in the first few frames is an artefact of re-orientating the image stack to align the embryo to the image axes (see Materials and Methods). Note the first, very faint oscillation occurring at 10:00 h. Time is indicated in hr:min.

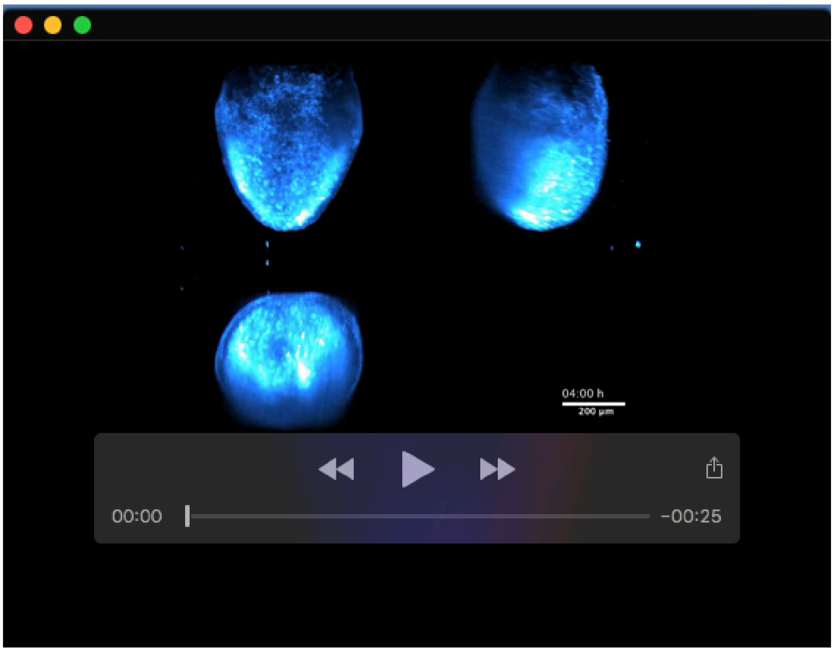

Movie 6. Dynamic activity patterns of knock-in reporter LfngT2A3xVenus before and during early somitogenesis. Related to Fig. S5. Venus fluorescence (cyan) is shown. The embryo was imaged from EB stage onwards from posterior side. The movie shows the embryo as MIP from three perspectives: posterior (top left), left side (top right), distal (bottom left). Note the strong autofluores-cence of visceral endoderm at the beginning of the experiment which partially masks the pulse in the mesoderm layer below. Time is indicated in hr:min.

Development • Supplementary information

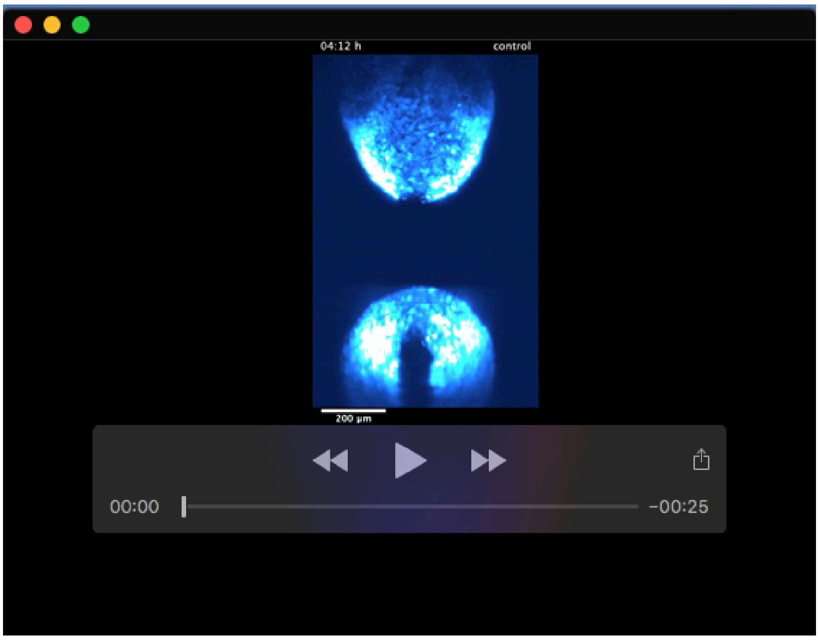

Movie 7. LuVeLu dynamics in control embryo. Related to Fig. 3. LuVeLu (cyan) fluorescence in a control embryo is shown from posterior (top) and distal orientation (bottom). Time is indicated in hr:min. The peak of the pulse (EB stage) is set at 00:00. The movie starts 48 min prior to the peak of the pulse.

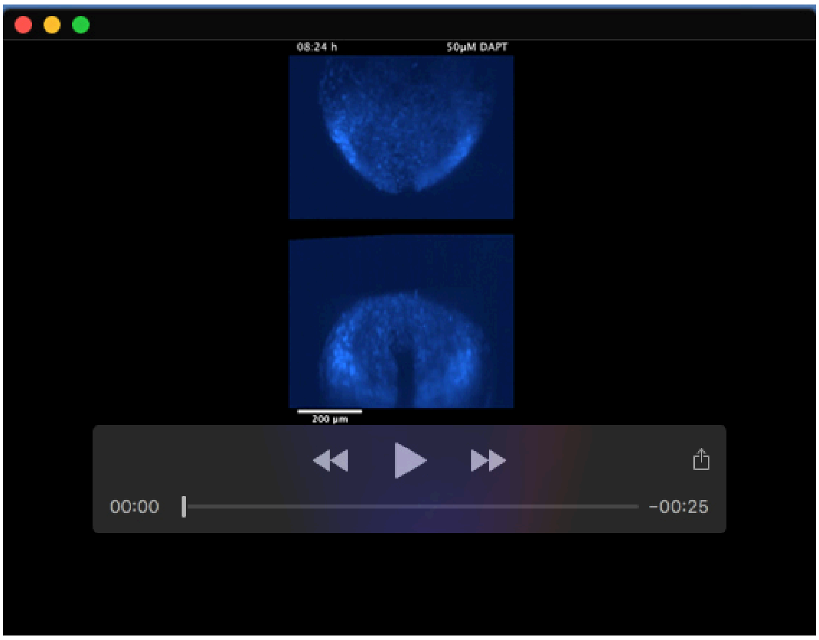

Movie 8. LuVeLu dynamics in DAPT treated embryo. Related to Fig. 3. LuVeLu (cyan) fluorescence in an embryo treated with 50 µM DAPT is shown from posterior (top) and distal orientation (bottom). Time is indicated in hr:min. The peak of the pulse (EB stage) is set at 00:00. The movie starts 48 min prior to the peak of the pulse, but DAPT treatment had started already 12 h earlier and was continued during imaging. Since embryos share the same culture medium on the light-sheet microscope, the control embryo (Movie 5) was imaged in a different experiment.

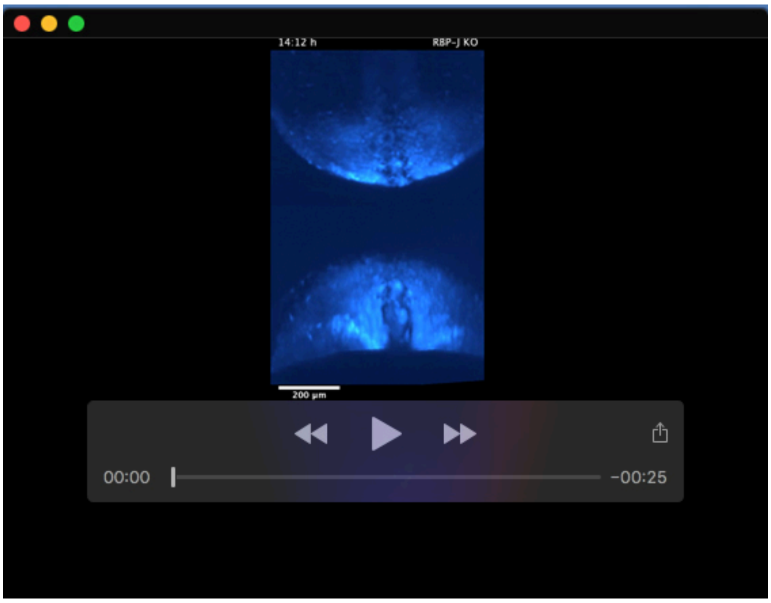

Movie 9. LuVeLu dynamics in *RBPjk*$^{-/-}$ embryos. Related to Fig. 3. LuVeLu (cyan) flu-orescence in *RBPjk*$^{-/-}$ embryo is shown from posterior (top) and distal orientation (bottom). Time is indicated in hr:min. The peak of the pulse (EB stage) is set at 00:00. The movie starts at the peak of the pulse.

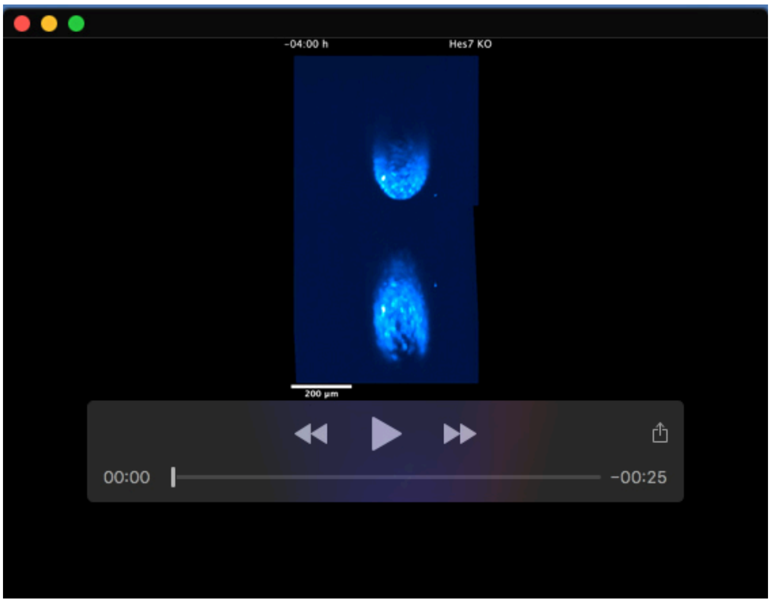

Movie 10. LuVeLu dynamics in *Hes7*$^{-/-}$ embryos. Related to Fig. 3. LuVeLu (cyan) fluores-cence in *Hes7*$^{-/-}$ embryo is shown from posterior (top) and distal orientation (bottom). Time is indicated in hr:min. The peak of the pulse (EB stage) is set at 00:00. The movie starts 8hr 24 min prior to the peak of the pulse.

Development • Supplementary information

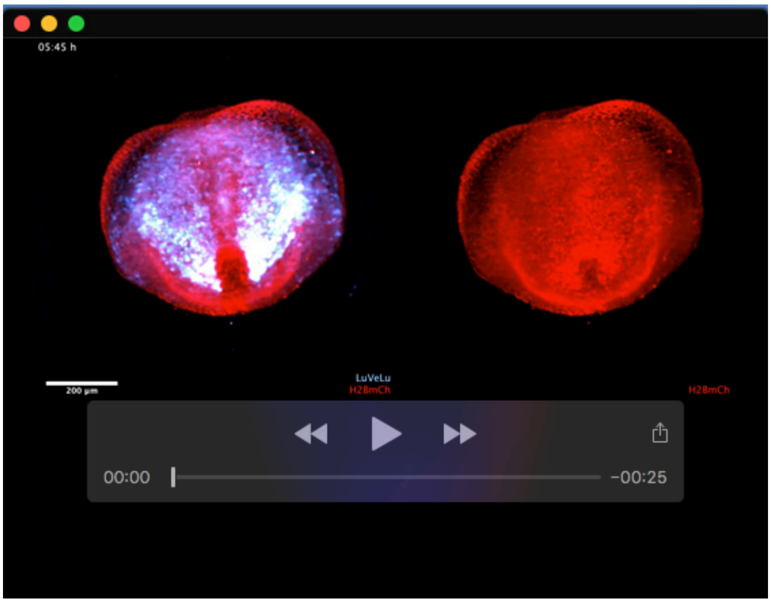

Movie 11. Somite boundary formation in relation to the first LuVeLu waves. Related to Fig. 4A–D. An embryo expressing the dynamic Notch signaling reporter LuVeLu (cyan) and nucleus marker R26-H2BmCherry (red) was imaged from late allantois bud stage onwards. To monitor the formation of somite boundaries, visible as clefts between nuclei, the embryo was mounted with the distal aspect toward the detection objective (posterior up). The same embryo with only the R26-H2BmCherry signal (red) is shown on the right, for better visualization of clefts. Time is indicated in hr:min.

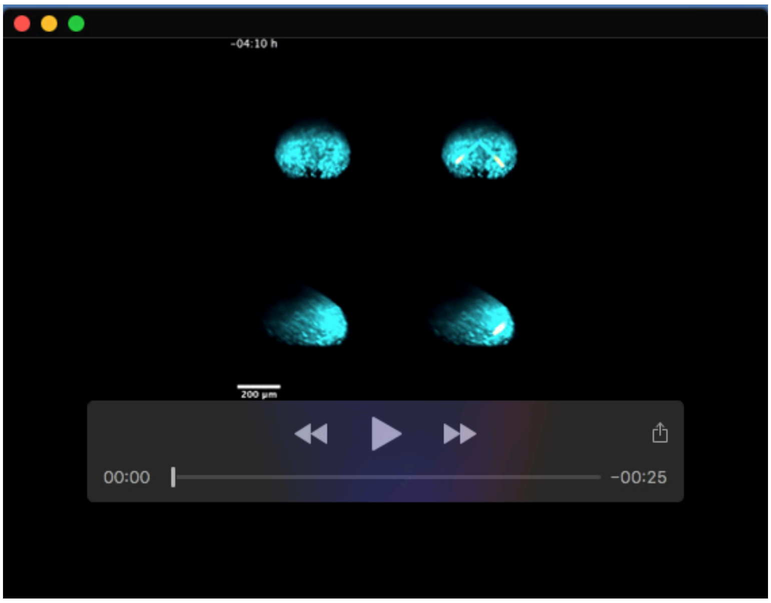

Movie 12. Line of interest used to generate surface kymographs from LuVeLu signal. Related to Fig. 5. LuVeLu (cyan) fluorescence in an embryo imaged from the early bud stage is shown from posterior (top) and side orientation (bottom). On the right, the line of interest used to generate a surface kymograph is overlaid. Time is indicated in hr:min.

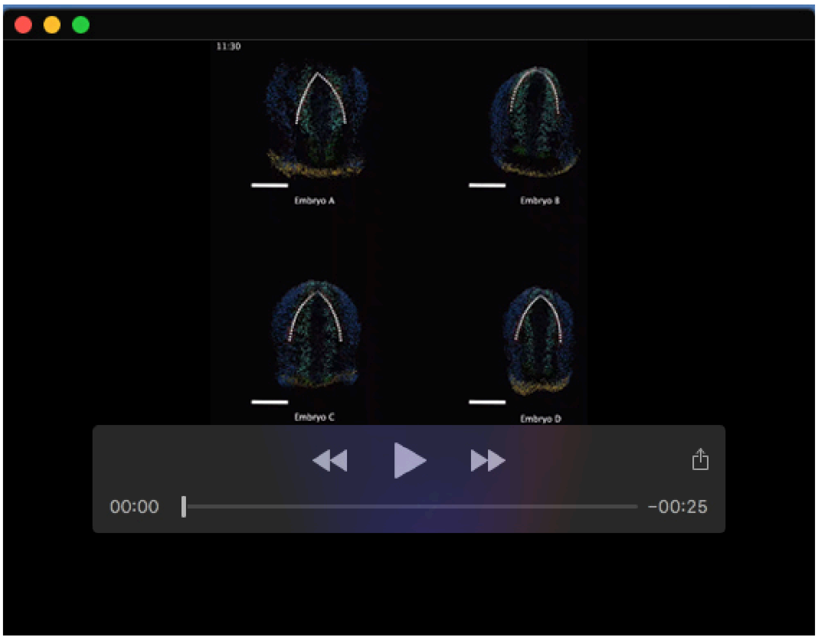

Movie 13. Transferred line of interest on cell tracking datasets. Related to Fig. 5C, Fig. S9. Cell locations in the cell tracking datasets are represented as octahedrons colorcoded with the following cell fate annotations : neural tube, somitic mesoderm, lateral plate mesoderm, anterior paraaxial mesoderm, and heart field/cardiogenic mesoderm. The transferred line of interest used to generate cell track/flow plots in Fig. 5C and Fig. S9 are represented as a line of white spheres. Cells within 30 µm of the line of interest are colored red. Time is indicated in hr:min, and the datasets are aligned so that 00:00 is set to the timepoint which corresponds to the start of the first wave. Scale bar: 200 µm.
